# Model and mesh selection from a mCRE functional in the context of parameter identification with full-field measurements

**DOI:** 10.1007/s00466-025-02598-1

**Published:** 2025-01-25

**Authors:** Hai Nam Nguyen, Ludovic Chamoin

**Affiliations:** 1 Le Quy Don Technical University, 236 Hoang Quoc Viet Street, Hanoi, 100000 Vietnam; 2 Université Paris-Saclay, CentraleSupélec, ENS Paris-Saclay, CNRS, LMPS - Laboratoire de Mécanique Paris-Saclay, 4 Avenue des Sciences, 91190 Gif-sur-Yvette, France

**Keywords:** Data assimilation, Constitutive relation error, Model identification, Model selection, Full-field measurements, Adaptive techniques, Model reduction

## Abstract

In this paper, we propose a general deterministic framework to question the relevance, assess the quality, and ultimately choose the features (in terms of model class and discretization mesh) of the employed computational mechanics model when performing parameter identification. The goal is to exploit both modeling and data at best, with optimized model accuracy and computational cost governed by the richness of available experimental information. Using the modified Constitutive Relation Error concept based on reliability of information and the construction of optimal admissible fields, we define rigorous quantitative error indicators that point out individual sources of error contained in the identified computational model with regards to (noisy) observations. An associated adaptive strategy is then proposed to automatically select, among a hierarchical list with increasing complexity, some parameterized mathematical model and finite element mesh which are consistent with the content of experimental data. In addition, the approach is computationally enhanced by the complementary use of model reduction techniques and specific nonlinear solvers. We focus here on experimental information given by full-field kinematic measurements, e.g. obtained by means of digital image correlation techniques, even though the proposed strategy would also apply to sparser data. The performance of the approach is analyzed and validated on several numerical experiments dealing with anisotropic linear elasticity or nonlinear elastoplastic models, and using synthetic or real observations.

## Introduction

Model identification and validation from experimental data has always been a fundamental engineering activity to ensure the fidelity of model-based numerical simulations compared to reality [78, 86]. This is particularly true for computational mechanics models which are commonly used into digital twins to analyze the response, perform diagnosis and prognosis, and eventually make decision on complex mechanical systems. Guaranteeing the consistency between outputs of such models and physical observations, through the identification of potentially complex constitutive laws and the calibration of additional model parameters, is nowadays a usual practice supported by advanced experimental techniques, such as digital image/volume correction (DIC/DVC) [46, 89] or optic fiber sensing [17, 66], which enable to collect rich data in terms of full-field kinematic measurements. In this data assimilation framework, the parametrized computational model employed for numerical simulation (with outputs compared to experimental data be solving an inverse problem) is usually chosen empirically, expecting sufficient accuracy to reproduce the observations. On the one hand, the class of the mathematical model (typically described by PDEs) is defined depending on *a priori* knowledge on the material/structure behavior and external environment, with a constitutive law picked among a list of physics-based laws with variable complexity (multi-fidelity modeling), and supposed to fit within a given parametric representation. On the other hand, the discretization attributes e.g., the finite element (FE) mesh used to describe the structural state, are set depending on the user experience. Consequently, the relevance of the chosen computational model with respect to (noisy) data is rarely questioned and verified in identification procedures. However, this looks as a natural concern when defining the identification problem; out of input parameters and data uncertainty, modeling uncertainty arises as a result of assumptions and simplifications. Modeling always remains an imperfect representation of the real world, and modeling errors are caused by all hypotheses and shortcuts taken while (i) translating physical phenomena into equations (mathematical model), and (ii) translating these equations into a form that can be solved numerically (discretization). They may have a large impact in inverse problems due to high sensitivity of the solution. Adaptive modeling, in terms of model class and discretization mesh, thus appears as a fundamental issue for effective inverse analysis and optimized identification process, trying to compute right at the right cost with a numerical complexity which is consistent with measurements at hand. Not only the model should be rich enough to perform a relevant interpretation of data and make full advantage of all experimental information available, in particular with full-field measurements, but it should not be too rich neither to avoid overfitting on always limited and noisy measurements, and thus prevent from useless computational effort. Therefore, the selection of the computational model should be smartly performed, adjusting its complexity and computational cost with respect to the physical content of experimental information. This is the topic of the present paper.

In the literature, quite few works dealing with parameter identification actually address the relevance of the computational model by considering, quantifying, and decreasing modeling error. These mainly place in the context of Bayesian inference [7, 15, 52, 65, 73, 84, 88] which offers a suitable stochastic framework to evaluate the plausibility of a given model class (within a set of concurrent classes) with respect to experimental data through the so-called model evidence, but this also goes with intensive computational resources. In the deterministic setting, much less work has been devoted to this task. It is mainly based on sensitivity analysis and refers to adaptive meshes, e.g. [5, 8, 24, 51, 85]. Specifically dealing with full-field measurements, model selection was addressed in [75] by using information from correlation residuals toward successive enrichments of the constitutive model in order to progressively reduce the experiment-model gap. In [64], mesh adaptivity was performed by using an empirical procedure based on sensitivity fields to define the mesh size. In [92], *p*-adaptive FE analysis was implemented in DIC to obtain a self-adapting higher order mesh capable of describing high-gradient displacement fields, here again without clear indicator on discretization error. In [47], a discretization error estimate was used to assess the quality of the mesh and relevance of prescribed boundary conditions (for a given constitutive model) in DIC/DVC computations. Out of computational mechanics, we mention the work reported in [3] that analyses the link between mesh density and measurement accuracy in optical diffusion tomography. Let us also mention a recent work, referred to as Efficient Unsupervised Constitutive Law Identification & Discovery (EUCLID) [34], which proposes a methodology to automatically select a constitutive model in a catalogue of material models from full-field displacements and global force data, without prescribing the structure of the constitutive law *a priori*. It shares common features with the present work, even though it only applies to full-field measurements (except in its supervised version designed for very specific scenarios [35]) and does not address data noise. Here, we wish to design a deterministic and automatic procedure that integrates measurement noise and modeling error into the identification procedure, in order to conveniently define the computational model and manage its reliability with respect to the richness of experimental information available.

The proposed work leans on the modified Constitutive Relation Error (mCRE) concept which has been initiated in [22, 56, 58] then used for decades for parameter identification, and which turns out to be a dedicated tool for the paper objectives. The mCRE concept refers to a deterministic variational inversion method that resorts to a primal-dual formulation [18] with Kohn-Vogelius-type functional [54], as an alternative to classical least square minimization. The approach is designed from the reliability of information and consists in splitting the overall (theoretical and experimental) knowledge on the inverse problem into two parts: (i) a reliable part e.g., balance equations or sensor locations, which defines an admissibility space and is strongly enforced in the inversion process; (ii) a complementary uncertain part e.g., constitutive law or measured data values, which is released and satisfied at best, thus accounting for model bias and measurement noise. This leads to a hybrid variational formulation with natural mix between measurement and modeling uncertainties, the optimal solution being obtained as a trade-off between modeling and experimental information. In other words, by releasing unreliable modeling information, the mCRE approach defines a data-based model enrichment, recovering the best state estimation (in terms of so-called admissible fields) from both mathematical model and experimental data; it thus naturally enters in the framework of hybrid twins as defined in [21]. Several attractive properties of the mCRE concept have been reported in the literature, such as the good convexity of its cost function with regularization from physics [2, 12], its capability to localize structural defects spatially [4, 13, 14, 29, 48] or its robustness to noisy or even corrupted measurements [9, 32].

The mCRE formulation has been applied in many contexts of computational mechanics, including forced vibrations [4, 26–29, 87], transient dynamics [13, 32], acoustics [25, 91], or joint/connexion identification [38, 79]. It was in particular used in association with image-based full-field measurements in [6, 9, 33, 41, 49] (and in a simplified version in [37, 40, 80]), showing advantages compared to alternative techniques such as the classical Finite Element Model Updating (FEMU) technique in terms of robustness and flexibility e.g., capability to integrate various levels of knowledge on boundary conditions.

In [77], a unified mCRE formulation was introduced when dealing with full-field measurements. Compared to previous works on the topic, additional ingredients were introduced to perform consistent parameter identification on a wide range of linear or nonlinear e.g., elasto-(visco-)plasticity, constitutive laws described by generalized standard material models with internal variables [45]. All *a priori* knowledge and uncertainty sources coming from model and data were integrated, connected, and propagated throughout the mCRE-based identification procedure using an appropriate metric, in order to make full benefit of information at hand. Nevertheless, even though the potential of mCRE to question the relevance of the employed computational model was shown, this potential was not used to its full extent.

In the present paper, we go one step further in this direction and in the convenience of using mCRE. We actually wish to extract more information from the terms that constitute the mCRE functional in order to conduct a smart definition of the identified computational model. In particular, it turns out that the Constitutive Relation Error (CRE) term of the mCRE acts as a modeling error estimator associated with the *a priori* chosen computational model. Its value thus naturally informs on the relevance of the identified model according to inferred data, and it may be split into local contributions over the whole physical domain. Starting from the formulation proposed in [77], we then further post-process the CRE term and develop some quantitative *a posteriori* error indicators on various modeling error sources (class of the parametrized constitutive law, discretization mesh) that identify the computational model inaccuracies with regards to experimental data. In particular, referring to CRE-based works on the verification of FE models (see [60, 62] for a review), it is shown that the indicator on discretization error requires the computation of a fully equilibrated stress field, which is easily obtained by post-processing the mCRE outputs. A sequential adaptive strategy driven by these indicators is then designed to assess error sources and select, in a greedy manner: (i) a suitable physics-based model (with appropriate physics) picked in a hierarchical list of model classes with increasing complexity and number of parameters; (ii) a suitable locally-refined space mesh. A related question is the definition of threshold values for the error indicators which permit to certify that the computational model is consistent with noisy data. The proposed methodology actually invokes a balance between modeling error and measurement noise, for full robustness and consistency. In addition, we introduce some advanced numerical techniques based on Proper Generalized Decomposition (PGD) model reduction [20] to decrease the computational effort of the proposed multi-query adaptive identification process. The performance of the approach is analyzed and validated on several numerical experiments dealing first with anisotropic linear elasticity models then nonlinear elastoplastic models, and using synthetic or real observations. Let us point out that even though we consider here full-field measurements, in order to better emphasize the impact of discretization error, the methodology also applies to any kind of experimental information, potentially obtained by more traditional sensing techniques (e.g. localized strain gauges), compared to the unsupervised version of the EUCLID framework for instance.

The paper is organized as follows: in Sect. 2, we introduce the context, notations, as well as basic concepts on the mCRE formulation for model identification purposes; the proposed mCRE-based modeling error estimation and the associated adaptive strategy used to perform model and mesh selection are developed in Sect. 3, together with advanced numerical techniques based on PGD to enhance computational efficiency; several numerical experiments are reported and analyzed in Sect. 4; eventually, conclusions and prospects to this research work are drawn in Sect. 5.

## Parameter identification from mCRE

### Reference problem and notations

**Fig. 1:**
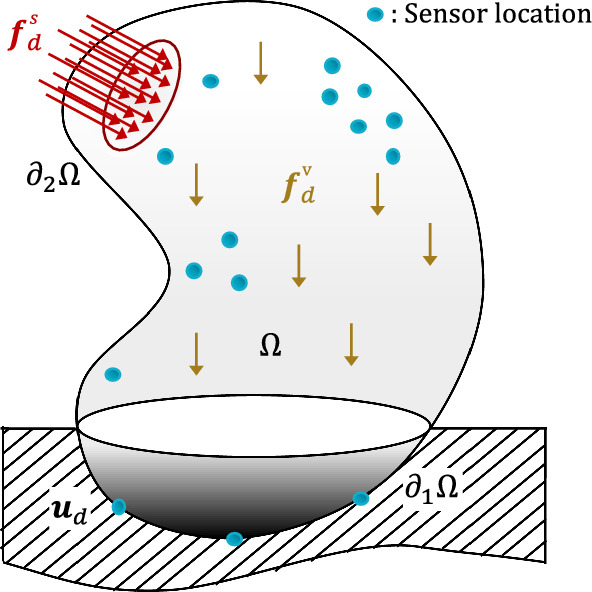
Configuration associated with the studied identification problem

We consider an open bounded domain $\varOmega \subset \mathbb {R}^d$ (*d* is the spatial dimension), with boundary $\partial \varOmega$, occupied by a deformable solid (Fig. 1). We assume that a displacement field ${\textbf{u}}_d$ is prescribed on part $\partial _1 \varOmega \subset \partial \varOmega$ of the boundary, and that tractions ${\textbf{f}}^s_d$ are prescribed on another part $\partial _2 \varOmega \subset \partial \varOmega$, with $\overline{\partial _1 \varOmega \cup \partial _2 \varOmega } = \partial \varOmega$ but potentially $\partial _1 \varOmega \cap \partial _2 \varOmega \ne \emptyset$. A body force field ${\textbf{f}}_d^v$ may also be given in $\varOmega$. Sufficient regularity is assumed for the prescribed data, that is ${\textbf{u}}_d \in [H^{1/2}(\partial _1 \varOmega )]^d$, ${\textbf{f}}^s_d \in [H^{-1/2}(\partial _2\varOmega )] ^d$, and ${\textbf{f}}_d \in [H^{-1}(\varOmega )]^d$. We assume small displacements, quasi-static loading, and isothermal conditions. To fix the ideas, we first consider a linear elastic material, even though nonlinear materials behaviors will be considered later. The direct problem (potentially ill-posed) associated with a displacement-stress solution pair  is then modeled by three groups of equations:

- kinematic admissibility, defining the space $\varvec{\mathcal {U}}_{ad}$ of compatible displacement fields satisfying Dirichlet boundary conditions:
  1 $$\begin{aligned} {\textbf{u}}\in [H^1(\varOmega )]^d \quad ; \quad {\textbf{u}}_{|\partial _1 \varOmega } = {\textbf{u}}_d \end{aligned}$$
- static admissibility, defining the space $\varvec{\mathcal {S}}_{ad}$ of $H(div,\varOmega )$ stress fields satisfying equilibrium equations written here in the weak form of the principle of virtual works: 2
- constitutive relation (Hooke’s law): 3

Tensors  and ${\textbf{K}}$ respectively denote the linearized strain tensor associated with the displacement ${\textbf{u}}$, and the symmetric positive definite Hooke tensor parametrized by a set ${\textbf{p}}$ of parameters (e.g., Young moduli, Poisson ratios, shear moduli, etc.) belonging to a parametric range space $\mathcal {P}$. The functional space $\varvec{\mathcal {U}}^{0,S}_{ad}$ is defined as:

4 $$\begin{aligned} \varvec{\mathcal {U}}^{0,S}_{ad} \equiv \left\{ {\textbf{v}}\in [H^1(\varOmega )]^d \, | \, {\textbf{v}}_{|\partial \varOmega \backslash \partial _2 \varOmega } = 0\right\} \end{aligned}$$

Denoting by $\varvec{\mathcal {U}}^0_{ad}$ the vectorial space associated with $\varvec{\mathcal {U}}_{ad}$, we have $\varvec{\mathcal {U}}^0_{ad} \subset \varvec{\mathcal {U}}^{0,S}_{ad}$.

We further consider the identification or updating of the set ${\textbf{p}}$ of unknown or only partially known scalar constitutive parameters. For this task, we assume that additional experimental information is available in terms of noisy observations ${\textbf{u}}_{m}$; these correspond here to full-field kinematic measurements which are indirectly obtained in a subregion of $\partial \varOmega$ or $\varOmega$ by means of DIC/DVC techniques.

### mCRE functional and inverse formulation

The mCRE formulation for inverse analysis is constructed from the CRE concept which introduces a computable measure of the residual on the constitutive relation (3) for any admissible pair . The CRE measure reads:5where $\Vert \bullet \Vert _{{\textbf{K}}^{-1}}$ stands for the energy norm on stress fields. This CRE measure has been widely used for model verification and mesh adaptation in FE simulations [60, 62].

Next, the mCRE approach for identification purposes is driven by the reliability of information, that is the *a priori* knowledge on model and measurements. It introduces a new admissibility space $({\textbf{A}}_d^-)$ in which all reliable information (equilibrium equations, known boundary conditions, etc.) is enforced. Other information (constitutive relation, noisy observation values, unknown boundary conditions, etc.) is then satisfied at best by minimizing a dedicated cost function. Considering for instance that only the constitutive relation and observation values are uncertain, we have $({\textbf{A}}_d^-)=\varvec{\mathcal {U}}_{ad} \times \varvec{\mathcal {S}}_{ad}$ and the mCRE functional is defined as:6with $\mathbb {G}_{obs}$ a normalizing matrix, ${\textbf{d}}$ the observation operator that evaluates the admissible displacement field ${\hat{{\textbf{u}}}}$ at sensing locations, and $\alpha$ a positive scalar weight. The definition of $\mathcal {E}_{mCRE}$ is of course flexible and can be adapted to other cases, such as uncertain loading ${\widetilde{{\textbf{f}}}}^s$ on a subregion of $\partial _2 {\widetilde{\varOmega }} \subset \partial _2 \varOmega$, to remain consistent with the reliability of information. In this latter case: (i) we would still take $({\textbf{A}}_d^-)=\varvec{\mathcal {U}}_{ad} \times \varvec{\mathcal {S}}_{ad}$ with ${\widetilde{{\textbf{f}}}}^s$ treated as an unknown field explicitly appearing in the definition (2) of $\varvec{\mathcal {S}}_{ad}$; (ii) an additional term of the form $\Vert {\widetilde{{\textbf{f}}}}^s-{\textbf{f}}^s_0\Vert ^2_{|\partial _2 {\widetilde{\varOmega }}}$ should be added in the mCRE functional, with ${\textbf{f}}^s_0$ a prior loading given by partial knowledge or by measurements.

**Fig. 2:**
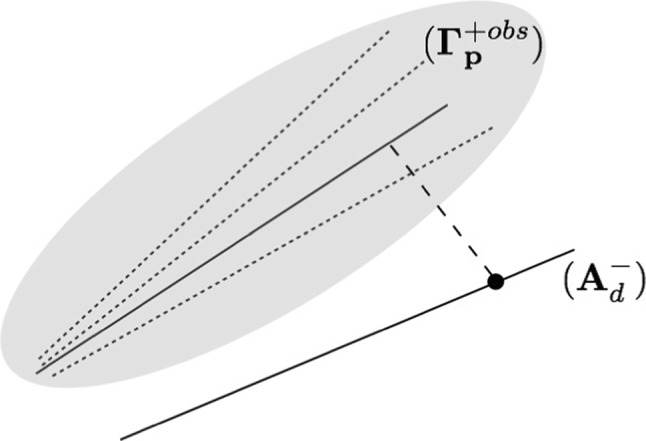
Geometrical interpretation of the mCRE strategy, with $({\textbf{A}}_d^-) \cap (\varvec{\varGamma }^{+ obs}_{\textbf{p}}) = \emptyset$

The inverse procedure thus reads:7We note that at the end of the process,  and the optimal admissible solution  satisfies a constitutive relation which is different from the *a priori* one (3), except in the idealistic case where admissibility conditions, measurement values and *a priori* constitutive relation are compatible one another. A geometrical interpretation of the mCRE-based inverse procedure is given in Fig. 2; it shows that a minimal distance (defined from the mCRE measure) is searched between the admissibility space $({\textbf{A}}_d^-)$ and the manifold $(\varvec{\varGamma }_{\textbf{p}}^{+obs})$ generated by the parametrized constitutive model (3) and the noisy observations. In most situations, $({\textbf{A}}_d^-) \cap (\varvec{\varGamma }_{\textbf{p}}^{+obs}) =\emptyset$.

The mCRE cost function (6) is made of two weighted terms which respectively refer to modeling and measurement gap terms. It can be seen as a classical least squares minimization (second term) regularized by physics (first term). The scalar weight $\alpha$, which has here the dimension of an energy, is then a parameter of the mCRE method that needs to be conveniently tuned in order to balance the two components of the mCRE functional properly. Limits correspond: (i) to the classical least squares minimization with prescribed constitutive relation and thus no modeling error taken into account *a priori*, when $\alpha \rightarrow 0$; (ii) to the pure CRE minimization with prescribed experimental data, when $\alpha \rightarrow \infty$. The value of $\alpha$ should be conveniently set, within these two limits, with regards to *a priori* quantitative information e.g., measurement noise level. Following the studies performed in [31, 68, 77], the value of $\alpha$ is here automatically set by referring to the Morozov discrepancy principle [71, 74]; $\alpha$ thus corresponds to the smallest value such that the final discrepancy between model outputs and observations falls within the measurement noise level. This has a clear physical sense, as it prevents from overfitting with meaningless decrease of the distance to measurements below the experimental uncertainty range.

When considering DIC-based full-field measurements, as it is the case in what follows, and denoting by ${\textbf{U}}_{DIC}$ the vector of nodal values of the measured field, the mCRE functional (6) is written as [77]:8with ${\textbf{d}}$ an operator that evaluates the kinematically admissible displacement field ${\hat{{\textbf{u}}}}$ (defined over the whole domain $\varOmega$) on nodes of the potentially local DIC mesh. The measurement gap term is here weighted by the inverse of the covariance matrix on measurement noise $\varSigma \hspace{-6.49994pt}{\varSigma }_{DIC}$, corresponding then to the Mahalanobis distance, and it is normalized with the number $N_{DIC}$ of measurement entries. Therefore, Morozov’s principle is satisfied when this weighted term is of the order of 1 (or equal to 1 statistically).

The discretized version of (8), using the FE method, reads:

9 $$\begin{aligned} \mathcal {E}^{h^2}_{mCRE}({\hat{{\textbf{U}}}},{\hat{{\textbf{V}}}};{\textbf{p}})  &   = \frac{1}{2}({\hat{{\textbf{U}}}}-{\hat{{\textbf{V}}}})^T\mathbb {K}({\textbf{p}})({\hat{{\textbf{U}}}}-{\hat{{\textbf{V}}}}) \nonumber \\  &   \quad + \frac{\alpha }{2}.\frac{1}{N_{DIC}}(\varPi {\hat{{\textbf{U}}}}-{\textbf{U}}_{DIC})^T\nonumber \\  &   \quad \varSigma \hspace{-6.49994pt}{\varSigma }_{DIC}^{-1}(\varPi {\hat{{\textbf{U}}}}-{\textbf{U}}_{DIC}) \end{aligned}$$

where $\varPi$ is an extraction-projection operator (usually a Boolean operator), while $\mathbb {K}$ denotes the global stiffness matrix. The vector ${\hat{{\textbf{V}}}}$ is associated with static admissibility and should satisfy the weak FEM version $\delta {\textbf{V}}^T\left( \mathbb {K}({\textbf{p}}){\hat{{\textbf{V}}}}-{\textbf{F}}\right) =0$ of balance equations (2), with ${\textbf{F}}$ the load vector, for any $\delta {\textbf{V}}\in \mathcal {U}_{ad,h}^{0,S}$. This results from duality arguments that indicate that the optimal statically admissible field  should stem from a displacement field ${\hat{{\textbf{v}}}}$ such that  [56].

#### Remark 1

A dimensionless mCRE functional may also be defined, under the form:10with ${\overline{\mathcal {E}}}^2_{mCRE} = \mathcal {E}^2_{mCRE}/\mathcal {E}^2_0$, ${\overline{\mathcal {E}}}^2_{CRE} = \mathcal {E}^2_{CRE}/\mathcal {E}^2_0$, and ${\tilde{\alpha }} = \alpha /\mathcal {E}^2_0$. The quantity $\mathcal {E}^2_0$ corresponds to a reference energy value; a typical choice is $\mathcal {E}^2_0 = \frac{1}{2}{\hat{{\textbf{U}}}}^{(0)^T}\mathbb {K}({\textbf{p}}^{(0)}){\hat{{\textbf{U}}}}^{(0)}$ where initial values of the parameter set (${\textbf{p}}^{(0)}$) and kinematic admissible field (${\hat{{\textbf{U}}}}^{(0)}$) are used. In the following, an alternative definition of the reference energy value is proposed to conduct the adaptive modeling algorithm.

### Practical implementation

The nonlinear constrained optimization problem (7) is in practice solved iteratively, with sequential partial minimization over admissibility and parameter spaces. The minimization is thus conducted with an iterative two-step algorithm in which, in each step: (i) optimal admissible fields are first computed by minimizing the mCRE functional under admissibility constraints, i.e. by finding the saddle point of a Lagrangian functional (see below), for fixed ${\textbf{p}}$; (ii) parameters ${\textbf{p}}$ are updated by implementing a gradient descent algorithm, the gradient being easily computed from the adjoint state method (and consequently from admissible fields obtained at the previous step). The strategy thus looks like an alternating direction strategy of block Gauss-Seidel type. It reads as follows:

0. Initialize the parameter set ${\textbf{p}}^{(0)}$.
  **Iteration loop (iteration **$n+1$)
1. Compute optimal admissible fields $({\hat{{\textbf{U}}}}^{(n+1)},{\hat{{\textbf{V}}}}^{(n+1)})$ for given ${\textbf{p}}^{(n)}$:
  11 $$\begin{aligned} ({\hat{{\textbf{U}}}}^{(n+1)},{\hat{{\textbf{V}}}}^{(n+1)}) = \mathop {\textrm{argmin}}\limits _{({\hat{{\textbf{U}}}},{\hat{{\textbf{V}}}})\in ({\textbf{A}}_d^-)}\mathcal {E}^{h^2}_{mCRE}({\hat{{\textbf{U}}}},{\hat{{\textbf{V}}}};{\textbf{p}}^{(n)}) \end{aligned}$$
2. Update ${\textbf{p}}^{(n)}$ with a gradient descent from $\mathcal {F}^h_{mCRE}({{\textbf{p}}}) = \mathcal {E}^{h^2}_{mCRE}({\hat{{\textbf{U}}}}^{(n+1)},{\hat{{\textbf{V}}}}^{(n+1)};{{\textbf{p}}})$:
  12 $$\begin{aligned} {\textbf{p}}^{(n+1)}={\textbf{p}}^{(n)} + \delta {\textbf{p}}^{(n)} \end{aligned}$$
3. If not converged, go to Step 1. Otherwise, stop.

Properties of the coupled system obtained in Step 1 play a fundamental role in the computational aspects of the mCRE minimization. It was shown that this specific system leads to a unique and stable solution (provided data is abundant enough) even in the case of missing information on boundary conditions for the direct problem. In addition, optimized numerical methods may be used to solve the coupled and potentially large system e.g., a (block) successive over-relaxation (SOR) technique was used in [4] in the case of large-scale inverse identification. Also, the Sherman-Morrison-Woodbury formula was used for system inversion in [68]. In Step 2 of the iterative algorithm, a backtracking line search (Armijo-Goldstein rule) may be used to set the step length in the gradient algorithm, but other methods (e.g. BFGS or Gauss-Newton) may also be used.

#### Remark 2

When the size of ${\textbf{p}}$ is large (e.g., when describing a parameter field), the spatial distribution of the (m)CRE cost function may be used to select and update only parameters that contribute most to the mismatch [26], in addition to detecting corrupted sensors. This is the so-called localization step, performed at the end of Step 1. The obtained hierarchical updating, with correction of parameters in zones with high local error alone, is associated with a minimization problem in a lower-dimension parameter space. It thus reduces the computational cost, and participates in the regularization process by guiding the identification/updating since naturally favoring an optimal configuration close to the initial one. Zones may correspond to sub-structures in engineering applications, or to finite elements when identifying a parameter field, and additional techniques such as clustering may be employed [31].

In order to enhance the numerical efficiency of the mCRE-based inversion procedure, and particularly the multi-query computation of optimal admissible fields $\left( {\hat{{\textbf{U}}}}({\textbf{p}}),{\hat{{\textbf{V}}}}({\textbf{p}})\right)$ in Step 1 for many values of the parameter set ${\textbf{p}}$ and of the scalar factor $\alpha$, PGD model reduction is of great interest. The idea is to compute, in an *offline phase*, a multi-parametrized optimal admissible solution that explicitly depends on ${\textbf{p}}$ and $\alpha$. This solution with modal structure can then be easily evaluated in the *online* stage when conducting the inversion process [16, 67]. An important feature is that PGD modes are computed from both model and experimental data, so that they integrate sensing information; this is in opposition to approaches where the PGD solution is constructed from the direct problem before being employed for inversion [42].

Giving some technical details, the constrained minimization performed in Step 1 of the mCRE minimization is based on the following Lagrangian functional (written in its FEM discretized form):

13 $$\begin{aligned} \mathcal {L}^h({\hat{{\textbf{U}}}},{\hat{{\textbf{V}}}},\varvec{\varLambda };{\textbf{p}})= &   \frac{1}{2}({\hat{{\textbf{U}}}}-{\hat{{\textbf{V}}}})^T\mathbb {K}({\textbf{p}})({\hat{{\textbf{U}}}}-{\hat{{\textbf{V}}}})\nonumber \\  &   + \frac{\alpha }{2}.\frac{1}{N_{DIC}}(\varPi {\hat{{\textbf{U}}}}-{\textbf{U}}_{DIC})^T\nonumber \\  &   \varSigma \hspace{-6.49994pt}{\varSigma }^{-1}_{DIC}(\varPi {\hat{{\textbf{U}}}}-{\textbf{U}}_{DIC})\nonumber \\  &   - \varvec{\varLambda }^T\left( \mathbb {K}({\textbf{p}}){\hat{{\textbf{V}}}}-{\textbf{F}}\right) \end{aligned}$$

where $\varvec{\varLambda }$ refers to nodal values of the Lagrange multiplier field. The first-order Karush-Kuhn-Tucker necessary optimality conditions, by searching the saddle-point of $\mathcal {L}^h$, read (subscript index *a* denotes active unknowns):

14 $$\begin{aligned}&\delta {\hat{{\textbf{U}}}}^T\left( \mathbb {K}({\textbf{p}})({\hat{{\textbf{U}}}}-{\hat{{\textbf{V}}}}) + \alpha .\frac{1}{N_{DIC}} \varPi ^T\varSigma \hspace{-6.49994pt}{\varSigma }^{-1}_{DIC}(\varPi {\hat{{\textbf{U}}}}-{\textbf{U}}_{DIC})\right) \nonumber \\&\quad = 0 \quad \forall \delta {\hat{{\textbf{U}}}} = [{\textbf{0}},\delta {\hat{{\textbf{U}}}}_a]^T \nonumber \\&\quad \delta {\hat{{\textbf{V}}}}^T\left( \mathbb {K}({\textbf{p}})({\hat{{\textbf{V}}}}-{\hat{{\textbf{U}}}}) - \mathbb {K}({\textbf{p}})\varvec{\varLambda }\right) = 0 \quad \forall \delta {\hat{{\textbf{V}}}} \nonumber \\&\quad \delta \varvec{\varLambda }^T\left( \mathbb {K}({\textbf{p}}){\hat{{\textbf{V}}}}-{\textbf{F}}\right) = 0 \quad \forall \delta \varvec{\varLambda }= [{\textbf{0}},\delta \varvec{\varLambda }_a]^T \end{aligned}$$

or, after substituting ${\hat{{\textbf{V}}}}$ ($={\hat{{\textbf{U}}}}+\varvec{\varLambda }$) from the second equation in (14):

15 $$\begin{aligned} \begin{aligned}&\delta {\hat{{\textbf{U}}}}^T\left( -\mathbb {K}({\textbf{p}})\varvec{\varLambda }+ \alpha .\frac{1}{N_{DIC}} \varPi ^T\varSigma \hspace{-6.49994pt}{\varSigma }^{-1}_{DIC}(\varPi {\hat{{\textbf{U}}}}-{\textbf{U}}_{DIC})\right) \\&\quad = 0 \quad \forall \delta {\hat{{\textbf{U}}}} = [{\textbf{0}},\delta {\hat{{\textbf{U}}}}_a]^T \\&\delta \varvec{\varLambda }^T\left( \mathbb {K}({\textbf{p}})({\hat{{\textbf{U}}}}+\varvec{\varLambda })-{\textbf{F}}\right) = 0 \quad \forall \delta \varvec{\varLambda }= [{\textbf{0}},\delta \varvec{\varLambda }_a]^T \end{aligned} \end{aligned}$$

This Galerkin formulation (in space) may be written in a more condensed form:

16 $$\begin{aligned} b\left( [{\hat{{\textbf{U}}}},\varvec{\varLambda }],[\delta {\hat{{\textbf{U}}}},\delta \varvec{\varLambda }]\right) = f\left( [\delta {\hat{{\textbf{U}}}},\delta \varvec{\varLambda }]\right) \quad \forall [\delta {\hat{{\textbf{U}}}},\delta \varvec{\varLambda }] \end{aligned}$$

with

17 $$\begin{aligned} \begin{aligned}&b\left( [{\hat{{\textbf{U}}}},\varvec{\varLambda }],[\delta {\hat{{\textbf{U}}}},\delta \varvec{\varLambda }]\right) = -\delta {\hat{{\textbf{U}}}}^T\mathbb {K}({\textbf{p}})\varvec{\varLambda }\\&\quad + \alpha .\frac{1}{N_{DIC}} \delta {\hat{{\textbf{U}}}}^T\varPi ^T\varSigma \hspace{-6.49994pt}{\varSigma }^{-1}_{DIC}\varPi {\hat{{\textbf{U}}}} + \delta \varvec{\varLambda }^T\mathbb {K}({\textbf{p}})({\hat{{\textbf{U}}}}+\varvec{\varLambda }) \\&f\left( [\delta {\hat{{\textbf{U}}}},\delta \varvec{\varLambda }]\right) = \alpha .\frac{1}{N_{DIC}} \delta {\hat{{\textbf{U}}}}^T\varPi ^T\varSigma \hspace{-6.49994pt}{\varSigma }^{-1}_{DIC} {\textbf{U}}_{DIC} + \delta \varvec{\varLambda }^T {\textbf{F}}\end{aligned} \end{aligned}$$

We thus implement the PGD model reduction technique by searching, in an *offline* phase, parametrized solutions $({\hat{{\textbf{U}}}},\varvec{\varLambda })$ with $\alpha$ and ${\textbf{p}}$ as extra-parameters. They are searched under the form:

18 $$\begin{aligned} {\hat{{\textbf{U}}}}_m(\alpha ,{\textbf{p}})  &   = \sum _{i=1}^m \left[ \varvec{\varPsi }^U_i \kappa _i^U(\alpha ) \prod _{j=1}^P \chi ^U_{j,i}(p_i) \right] \nonumber \\  &   \quad ; \quad \varvec{\varLambda }_m(\alpha ,{\textbf{p}}) = \sum _{i=1}^m \left[ \varvec{\varPsi }^\varLambda _i \kappa _i^\varLambda (\alpha ) \prod _{j=1}^P \chi ^\varLambda _{j,i}(p_i) \right] \nonumber \\ \end{aligned}$$

The conventional progressive Galerkin approach (e.g., described in [20]) is then implemented with the following global bilinear form *B* and linear form *F*:

19 $$\begin{aligned}  &   B\left( [{\hat{{\textbf{U}}}},\varvec{\varLambda }],[\delta {\hat{{\textbf{U}}}},\delta \varvec{\varLambda }]\right) = \int _{\varOmega _\alpha }\int _{\mathcal {P}} b\left( [{\hat{{\textbf{U}}}},\varvec{\varLambda }],[\delta {\hat{{\textbf{U}}}},\delta \varvec{\varLambda }]\right) \quad ;\nonumber \\  &   \quad F\left( [\delta {\hat{{\textbf{U}}}},\delta \varvec{\varLambda }]\right) = \int _{\varOmega _\alpha }\int _{\mathcal {P}} f\left( [\delta {\hat{{\textbf{U}}}},\delta \varvec{\varLambda }]\right) \nonumber \\ \end{aligned}$$

with $\varOmega _\alpha = \mathbb {R}^{*+}$ and $\mathcal {P}$ the spaces of parameters $\alpha$ and ${\textbf{p}}$, respectively.

The PGD solutions ${\hat{{\textbf{U}}}}_m(\alpha ,{\textbf{p}})$ and ${\hat{{\textbf{V}}}}_m(\alpha ,{\textbf{p}}) = {\hat{{\textbf{U}}}}_m(\alpha ,{\textbf{p}}) + \varvec{\varLambda }_m(\alpha ,{\textbf{p}})$, with explicit dependency on ${\textbf{p}}$ and $\alpha$, are then used in the *online* model updating phase with mCRE, allowing for: (i) fast evaluation of optimal admissible fields for any value of ${\textbf{p}}$, and therefore fast computation of gradients of the mCRE cost function in Step 2; (ii) simplified search of the optimal value of $\alpha$, satisfying the Morozov principle.

### Extension to nonlinear materials

We now briefly present the natural extension of the mCRE concept to nonlinear dissipative materials (with standard formulation) evolving in a domain $\varOmega$ over a time interval [0, *T*], as developed in [68, 77]. It mainly refers to a general definition of the CRE measure using Legendre-conjugate dual convex potentials defined by the thermodynamics of continua (see [57]), when considering generalized standard material models with internal variables [39, 45]. This general CRE definition is also strongly linked to the symmetrized Bregman divergence [19] .

In this framework, we define global flux variables ,  (with ), where  (resp. ) is the elastic (resp. plastic) part of the total strain , while ${\textbf{X}}$ gathers additional internal variables such as cumulative plastic strain. We also define associated global force variables  where ${\textbf{Y}}$ gathers thermodynamic forces associated with internal variables in ${\textbf{X}}$.

The constitutive behavior is then basically described by two complementary sets of equations:

- state equations, derived from the Helmholtz free energy $\psi$ which is a positive and convex potential:
  20 $$\begin{aligned} {\textbf{s}}=\frac{\partial \psi }{\partial {\textbf{e}}_e} \quad \text {or} \quad {\textbf{e}}_e=\frac{\partial \psi ^*}{\partial {\textbf{s}}} \end{aligned}$$
  $\psi ^*$ is the dual potential of $\psi$, defined from the Legendre-Fenchel transform [70]:
  21 $$\begin{aligned} \psi ^*({\textbf{s}}) = \sup _{{\textbf{e}}_e}\left[ {\textbf{s}}\cdot {\textbf{e}}_e - \psi ({\textbf{e}}_e)\right] \end{aligned}$$
  In the linear elasticity case developed previously, potentials $\psi$ and $\psi ^*$ are quadratic and read  and .
- evolution laws, derived from a dissipation potential $\varphi$:
  22 $$\begin{aligned} {\textbf{s}}= \frac{\partial \varphi }{\partial {\dot{{\textbf{e}}}}_p} \quad \text {or} \quad {\dot{{\textbf{e}}}}_p = \frac{\partial \varphi ^*}{\partial {\textbf{s}}} \end{aligned}$$
  where $\varphi ^*$ is the Legendre-conjugate dual potential of $\varphi$:
  23 $$\begin{aligned} \varphi ^*({\textbf{s}}) = \sup _{{\dot{{\textbf{e}}}}_p}\left[ {\textbf{s}}\cdot {\dot{{\textbf{e}}}}_p - \varphi ({\dot{{\textbf{e}}}}_p)\right] \end{aligned}$$
  Choosing a convex, non-negative and zero at origin potential $\varphi$ is a sufficient condition to satisfy the Clausius-Duhem inequality with intrinsic dissipation $\varPhi \equiv {\textbf{s}}\cdot {\dot{{\textbf{e}}}}_p \ge 0$.

A consistent CRE measure can then be defined as:

24 $$\begin{aligned} \mathcal {E}^2_{CRE}({\hat{{\textbf{e}}}},{\hat{{\textbf{s}}}}) = \int _0^T\int _{\varOmega }\left( \eta _\psi ({\hat{{\textbf{e}}}}_e,{\hat{{\textbf{s}}}}) + \int _0^t \eta _\varphi (\dot{{\hat{{\textbf{e}}}}}_p,{\hat{{\textbf{s}}}})\right) \ge 0\nonumber \\ \end{aligned}$$

with

25 $$\begin{aligned}  &   \eta _\psi ({\hat{{\textbf{e}}}}_e,{\hat{{\textbf{s}}}}) \equiv \psi ({\hat{{\textbf{e}}}}_e)+\psi ^*({\hat{{\textbf{s}}}})- {\hat{{\textbf{s}}}} \cdot {\hat{{\textbf{e}}}}_e \ge 0 \quad ; \quad \nonumber \\  &   \eta _\varphi (\dot{{\hat{{\textbf{e}}}}}_p,{\hat{{\textbf{s}}}}) \equiv \varphi (\dot{{\hat{{\textbf{e}}}}}_p)+\varphi ^*({\hat{{\textbf{s}}}})- {\hat{{\textbf{s}}}} \cdot \dot{{\hat{{\textbf{e}}}}}_p \ge 0 \end{aligned}$$

corresponding to the residuals on state equations and evolution laws, respectively, which are local in space and time quantities. This CRE measure is associated with an admissible solution $({\hat{{\textbf{e}}}}_e,{\hat{{\textbf{e}}}}_p,{\hat{{\textbf{s}}}})$ such that ${\hat{{\textbf{e}}}}_e+{\hat{{\textbf{e}}}}_p={\hat{{\textbf{e}}}}$, and it vanishes when constitutive relations (20) and (22) are satisfied at any space-time point.

From the definition (24) of the CRE measure, a direct and natural extension of the mCRE functional to generalized standard materials is proposed. It reads when coupled with DIC measurements:

26 $$\begin{aligned} \mathcal {E}^2_{mCRE}({\hat{{\textbf{e}}}},{\hat{{\textbf{s}}}};{\textbf{p}})= &   \mathcal {E}^2_{CRE}({\hat{{\textbf{e}}}},{\hat{{\textbf{s}}}};{\textbf{p}}) \nonumber \\  &   + \frac{\alpha }{2}.\frac{1}{N_{DIC}.N_t}\sum _{n_t=1}^{N_t}\left( {\textbf{d}}({\hat{{\textbf{u}}}}^{n_t})-{\textbf{U}}^{n_t}_{DIC}\right) ^T\nonumber \\    &   \varSigma \hspace{-6.49994pt}{\varSigma }_{DIC}^{-1}\left( {\textbf{d}}({\hat{{\textbf{u}}}}^{n_t})-{\textbf{U}}^{n_t}_{DIC}\right) \ \end{aligned}$$

where $N_t$ denotes the number of data assimilation time points. As it is naturally derived from the thermodynamics framework, this functional keeps advantages associated with convexity properties. It is applied to an admissible solution in $({\textbf{A}}_d^-)$, that should now also include initial conditions.

As indicated previously, the solution to the inverse problem corresponds to the parameter set ${\textbf{p}}_{sol}$ that satisfies the following nested minimization:

27 $$\begin{aligned} {\textbf{p}}^{sol} = \mathop {\textrm{argmin}}\limits _{{\textbf{p}}\in \mathcal {P}}\left[ \min _{({\hat{{\textbf{e}}}},{\hat{{\textbf{s}}}})\in ({\textbf{A}}_d^-)}\mathcal {E}^2_{mCRE}({\hat{{\textbf{e}}}},{\hat{{\textbf{s}}}};{\textbf{p}})\right] \end{aligned}$$

It is again solved by means of an alternated minimization scheme in which, at iteration $k+1$:

- Step 1: an optimal admissible set $({\hat{{\textbf{e}}}}^{(k+1)},{\hat{{\textbf{s}}}}^{(k+1)})$ is searched by solving the first partial minimization over $({\textbf{A}}_d^-)$, for given ${\textbf{p}}={\textbf{p}}^{(k)}$:
  28 $$\begin{aligned} ({\hat{{\textbf{e}}}}^{(k+1)}{,} {\hat{{\textbf{s}}}}^{(k+1)}) = \mathop {\textrm{argmin}}\limits _{({\hat{{\textbf{e}}}},{\hat{{\textbf{s}}}})\in ({\textbf{A}}_d^-)}\mathcal {E}^2_{mCRE}({\hat{{\textbf{e}}}},{\hat{{\textbf{s}}}};{\textbf{p}}^{(k)}) \end{aligned}$$
- Step 2: a new parameter set ${\textbf{p}}^{(k+1)}$ is computed by solving the second partial minimization over $\mathcal {P}$, for the optimal admissible fields obtained at Step 1:
  29 $$\begin{aligned} {\textbf{p}}^{(k+1)} = \mathop {\textrm{argmin}}\limits _{{\textbf{p}}\in \mathcal {P}}\mathcal {E}^2_{mCRE}({\hat{{\textbf{e}}}}^{(k+1)}_e,{\hat{{\textbf{e}}}}^{(k+1)}_p,{\hat{{\textbf{s}}}}^{(k+1)};{\textbf{p}}) \end{aligned}$$

**Fig. 3:**
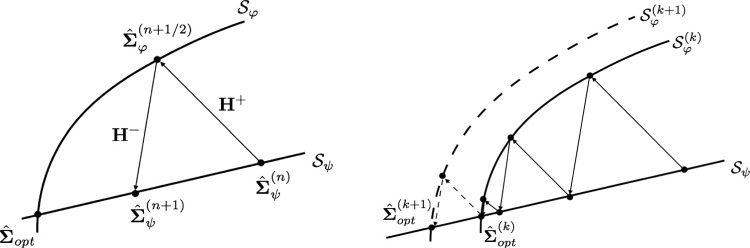
Graphical sketch of the algorithm used in the mCRE framework with nonlinear dissipative material behaviors (left), and of the multi-resolution procedure (right)

In practice, as detailed in [77], a dedicated iterative non-incremental solver is used to perform the constrained minimization (28) of Step 1 which is the most expensive and technical one for nonlinear models. Sharing similarities with the LATIN method proposed in [59], with a splitting between linear (potentially global in space) equations and local in space (potentially nonlinear) equations, the solver effectively addresses material nonlinearities in the inverse problem. For this purpose, the mCRE functional defined in (26) is split into two positive parts:

30 $$\begin{aligned} \mathcal {E}^2_{mCRE}({\hat{{\textbf{e}}}}_e,{\hat{{\textbf{e}}}}_p,{\hat{{\textbf{s}}}};{\textbf{p}}) = \mathcal {E}^2_\psi ({\hat{{\textbf{e}}}}_e,{\hat{{\textbf{e}}}}_p,{\hat{{\textbf{s}}}};{\textbf{p}}) + \mathcal {E}^2_\varphi ({\hat{{\textbf{e}}}}_e,{\hat{{\textbf{e}}}}_p,{\hat{{\textbf{s}}}};{\textbf{p}})\nonumber \\ \end{aligned}$$

with

31 $$\begin{aligned} \mathcal {E}^2_\psi ({\hat{{\textbf{e}}}}_e,{\hat{{\textbf{e}}}}_p,{\hat{{\textbf{s}}}};{\textbf{p}})&\equiv \int _0^T\int _{\varOmega }\eta _\psi ({\hat{{\textbf{e}}}}_e,{\hat{{\textbf{s}}}};{\textbf{p}}) \nonumber \\&\quad + \frac{\alpha }{2}.\frac{1}{N_{DIC}.N_t}\sum _{n_t=1}^{N_t}\left( {\textbf{d}}({\hat{{\textbf{u}}}}^{n_t})-{\textbf{U}}^{n_t}_{DIC}\right) ^T\nonumber \\  &\quad \varSigma \hspace{-6.49994pt}{\varSigma }_{DIC}^{-1}\left( {\textbf{d}}({\hat{{\textbf{u}}}}^{n_t})-{\textbf{U}}^{n_t}_{DIC}\right) \nonumber \\ \mathcal {E}^2_\varphi ({\hat{{\textbf{e}}}}_e,{\hat{{\textbf{e}}}}_p,{\hat{{\textbf{s}}}};{\textbf{p}})&\equiv \int _0^T\int _{\varOmega }\int _0^t \eta _\varphi (\dot{{\hat{{\textbf{e}}}}}_p,{\hat{{\textbf{s}}}};{\textbf{p}}) \end{aligned}$$

Next, we introduce variable sets ${\hat{\varvec{\varSigma }}}_\psi$ and ${\hat{\varvec{\varSigma }}}_\varphi$ which correspond to the original variable set ${\hat{\varvec{\varSigma }}}=({\hat{{\textbf{e}}}},{\hat{{\textbf{s}}}})$ in which some variables are frozen and others are let free. They are such that:

- in ${\hat{\varvec{\varSigma }}}_\psi$, all internal variables driven by evolution laws (e.g. the plastic strain ) are frozen; only ${\textbf{u}}$ (or ) and  are free;
- in ${\hat{\varvec{\varSigma }}}_\varphi$, the total strain  is frozen while other variables appearing in the dissipation are free.

These two sets enable to reformulate the initial constrained minimization problem as two coupled minimizations in reduced spaces:

32 $$\begin{aligned} \min _{{\hat{\varvec{\varSigma }}}_\psi \in ({\textbf{A}}_d^{-,\psi })}\mathcal {E}^2_\psi ({\hat{\varvec{\varSigma }}}_\psi ;{\textbf{p}}) \quad ; \quad \min _{{\hat{\varvec{\varSigma }}}_\varphi \in ({\textbf{A}}_d^{-,\varphi })}\mathcal {E}^2_\varphi ({\hat{\varvec{\varSigma }}}_\varphi ;{\textbf{p}}) \end{aligned}$$

In this framework:

- The constraint ${\hat{\varvec{\varSigma }}}_\psi \in ({\textbf{A}}_d^{-,\psi })$ involves equilibrium equations and boundary conditions. The constrained minimization of $\mathcal {E}^2_\psi$, using Lagrange multiplier fields, is thus associated with a problem which is global over the whole space-time domain but which is linear. The solution manifold corresponds to the linear space $\mathcal {S}_\psi \equiv \{\mathop {\textrm{argmin}}\limits _{{\hat{\varvec{\varSigma }}}_\psi \in ({\textbf{A}}_d^{-,\psi })}\mathcal {E}^2_\psi ({\hat{\varvec{\varSigma }}}_\psi ;{\textbf{p}})\}$.
- The constraint ${\hat{\varvec{\varSigma }}}_\varphi \in ({\textbf{A}}_d^{-,\varphi })$ involves initial conditions alone. The constrained minimization of $\mathcal {E}^2_\varphi$ is thus associated with a problem which is local in space (and global in time), but which is nonlinear. The solution manifold corresponds to the nonlinear space $\mathcal {S}_\varphi \equiv \{\mathop {\textrm{argmin}}\limits _{{\hat{\varvec{\varSigma }}}_\varphi \in ({\textbf{A}}_d^{-,\varphi })}\mathcal {E}^2_\varphi ({\hat{\varvec{\varSigma }}}_\varphi ;{\textbf{p}})\}$.

The solution scheme thus consists in alternating solutions of a local stage (nonlinear and local in space problem) and a linear stage (linear and global in space problem), each being global in time and leading to an approximate solution of the optimal admissible pair $({\hat{{\textbf{e}}}},{\hat{{\textbf{s}}}}) = \mathcal {S}_\psi \cap \mathcal {S}_\varphi$ (for given ${\textbf{p}}$) over the whole space-time interval. After introducing up and down directions ${\textbf{H}}^+$ and ${\textbf{H}}^-$, respectively, the local and linear stages are performed as follows:

- In the local stage, internal variables are searched at the integration point level, so that calculations can be parallelized: given ${\hat{\varvec{\varSigma }}}^{(n)}_\psi \in \mathcal {S}_\psi$, find ${\hat{\varvec{\varSigma }}}^{(n+1/2)}_\varphi \in \mathcal {S}_\varphi$ such that:
  33 $$\begin{aligned} ({\hat{\varvec{\varSigma }}}^{(n+1/2)}_\varphi -{\hat{\varvec{\varSigma }}}^{(n)}_\psi ) \in {\textbf{H}}^+ \end{aligned}$$
  Among the various choices for ${\textbf{H}}^+$, we consider here the one that prescribes the same strain tensor, that is  (so-called *infinity direction* in the literature, with vertical slope). This way, .
- In the linear stage, the following global in space problem is solved: given ${\hat{\varvec{\varSigma }}}^{(n+1/2)}_\varphi \in \mathcal {S}_\varphi$, find ${\hat{\varvec{\varSigma }}}^{(n+1)}_\psi \in \mathcal {S}_\psi$ such that:
  34 $$\begin{aligned} ({\hat{\varvec{\varSigma }}}^{(n+1)}_\psi -{\hat{\varvec{\varSigma }}}^{(n+1/2)}_\varphi ) \in {\textbf{H}}^- \end{aligned}$$
  The optimal choice for ${\textbf{H}}^-$ is the local tangent direction to $\mathcal {S}_\varphi$, similarly to a classical Newton method, but this requires to compute a tangent operator at each iteration. For the sake of simplicity, we rather consider an elastic down direction that relates variations in stress and elastic strain tensors.

An illustration of the proposed LATIN-like algorithm within the mCRE framework is shown in Fig. 3.

We also mention that over the whole iterative mCRE minimization process, we can fully make benefit of the multi-resolution aspect of the LATIN algorithm, with restart procedure [76]. Once material parameters ${\textbf{p}}$ are updated, the previously computed space-time solution (in terms of optimal admissible fields obtained with ${\textbf{p}}^{(k)}$) is in practice reused as the initialization of another iterative procedure with the new set ${\textbf{p}}^{(k+1)}$ of material parameters (Fig. 3).

## Modeling error estimation and adaptive identification process

In this section, we question the quality of the computational model used for identification by introducing appropriate error estimation and adaptivity tools. For the sake of conciseness, we develop the methodology by coming back to a linear elasticity model, i.e. by using the mCRE functional defined in (5) and (8). Nevertheless, the proposed methodology applies the same way to nonlinear dissipative models using the general mCRE functional defined in (24) and (26).

### Error indicator on the model class

Until now, and in most works of the literature, the mCRE framework has been used in association with a fixed model class, i.e. material behavior model, chosen *a priori* from subjective knowledge and supposed to fit within a given parametric representation. We show below that the mCRE formulation actually permits to quantify the relevance of this model class with respect to experimental observations. This refers to the stochastic interpretation of the mCRE metric given in [27] in the Bayesian inference context with maximum a posteriori (MAP) estimator. As an alternative to considering modeling error at measurement points by means of a covariance matrix (which is in practice usually poorly known if not neglected), the mCRE strategy integrates modeling error in a global manner. It comes with a probability density function, incorporated in the likelihood function, that quantitatively reflects the confidence on modeling.

**Fig. 4:**
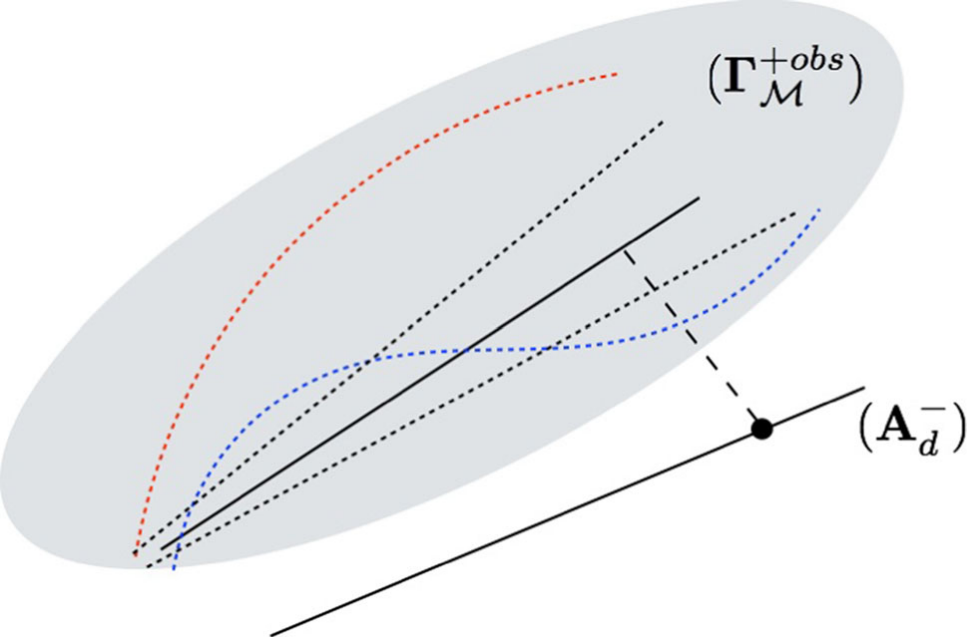
Geometrical illustration of the proposed model selection strategy from mCRE

The idea is thus to let the model class free, searching the optimal class iteratively from information contained in the computed quantity $\mathcal {E}^{h^2}_{mCRE}({\hat{{\textbf{U}}}}^{sol},{\hat{{\textbf{V}}}}^{sol};{\textbf{p}}^{sol})$ . The associated strategy is geometrically represented in Fig. 4, as a substitute of Fig. 2. We now deal with a manifold $(\varvec{\varGamma }_\mathcal {M}^{+obs})$ of possible models with choice on the model class $\mathcal {M}$, in practice among a list of parametrized constitutive models with hierarchic complexity.

In order to define a quantitative criterion for adaptive modeling, we first analyze the idealistic scenario where the outputs of the identified linear elasticity model perfectly reproduce the exact physical quantities, that is $\varPi {\textbf{U}}= {\textbf{U}}_{phys}$ where ${\textbf{U}}$ is the (admissible) solution of the direct problem $\mathbb {K}({\textbf{p}}^{sol}){\textbf{U}}={\textbf{F}}$. In such a scenario, the identified model is compatible with physics so that there is no modeling error. We also note that in the discretized mCRE framework, the optimal admissible field ${\hat{{\textbf{V}}}}$ (for given ${\textbf{p}}$) corresponds to a kinematically admissible field of the form $[{\textbf{U}}_d,{\hat{{\textbf{V}}}}_a]$ as ${\hat{{\textbf{V}}}}={\hat{{\textbf{U}}}}+\varvec{\varLambda }$ up to a rigid body displacement field which is considered null; it further satisfies $\mathbb {K}({\textbf{p}}){\hat{{\textbf{V}}}}={\textbf{F}}$. Consequently, for ${\textbf{p}}={\textbf{p}}^{sol}$, ${\hat{{\textbf{V}}}}^{sol}={\textbf{U}}$.

In the hypothetic case of no measurement noise, DIC measurements exactly correspond to the outputs of the updated model $\mathbb {K}({\textbf{p}}^{sol}){\textbf{U}}={\textbf{F}}$, i.e. ${\textbf{U}}_{DIC} = \varPi {\textbf{U}}$ ($= \varPi {\hat{{\textbf{V}}}}^{sol}$ when ${\textbf{p}}={\textbf{p}}^{sol}$). Therefore, at convergence of the mCRE minimization, the system describing the optimal admissible fields and obtained at Step 1 of the iterative mCRE strategy reads:

35 $$\begin{aligned}&\left[ \mathbb {K}_{aa}({\textbf{p}}^{sol}) + \alpha .\frac{1}{N_{DIC}} (\varPi ^T \varSigma \hspace{-6.49994pt}{\varSigma }^{-1}_{DIC}\varPi )_{aa}\right] {\hat{{\textbf{U}}}}^{sol}_a = {\textbf{F}}_a \nonumber \\&\quad + \alpha .\frac{1}{N_{DIC}} (\varPi ^T \varSigma \hspace{-6.49994pt}{\varSigma }^{-1}_{DIC})_{ao}{\textbf{U}}_{DIC}\nonumber \\&\quad - \mathbb {K}_{ad}({\textbf{p}}^{sol}){\textbf{U}}_d -\alpha .\frac{1}{N_{DIC}} (\varPi ^T\varSigma \hspace{-6.49994pt}{\varSigma }^{-1}_{DIC}\varPi )_{ad}{\textbf{U}}_d \nonumber \\&\quad = \mathbb {K}_{ao}({\textbf{p}}^{sol}){\hat{{\textbf{V}}}}^{sol} + \alpha .\frac{1}{N_{DIC}} (\varPi ^T \varSigma \hspace{-6.49994pt}{\varSigma }^{-1}_{DIC}\varPi )_{ao}{\hat{{\textbf{V}}}}^{sol} \nonumber \\&\quad - \mathbb {K}_{ad}({\textbf{p}}^{sol}){\textbf{U}}_d -\alpha .\frac{1}{N_{DIC}} (\varPi ^T\varSigma \hspace{-6.49994pt}{\varSigma }^{-1}_{DIC}\varPi )_{ad}{\textbf{U}}_d \nonumber \\&\quad = \left[ \mathbb {K}_{aa}({\textbf{p}}^{sol}) + \alpha .\frac{1}{N_{DIC}} (\varPi ^T \varSigma \hspace{-6.49994pt}{\varSigma }^{-1}_{DIC}\varPi )_{aa}\right] {\hat{{\textbf{V}}}}^{sol}_a \end{aligned}$$

so that ${\hat{{\textbf{U}}}}^{sol}_a={\hat{{\textbf{V}}}}^{sol}_a$, and consequently ${\hat{{\textbf{U}}}}^{sol}={\hat{{\textbf{V}}}}^{sol}$ ($={\textbf{U}}$). Therefore, this naturally yields $\mathcal {E}^{h^2}_{mCRE}({\hat{{\textbf{U}}}}^{sol},{\hat{{\textbf{V}}}}^{sol};{\textbf{p}}^{sol})=0$ with the two mCRE components including $\mathcal {E}^{h^2}_{CRE}({\hat{{\textbf{U}}}}^{sol},{\hat{{\textbf{V}}}}^{sol};{\textbf{p}}^{sol})$ that vanish for any value of $\alpha$.

When now considering DIC measurement noise, still with an exact model, then ${\textbf{U}}_{DIC}=\varPi {\hat{{\textbf{V}}}}^{sol} + \varvec{\xi }$ for ${\textbf{p}}={\textbf{p}}^{sol}$, with $\varvec{\xi }$ a random vector with zero mean and covariance $\varSigma \hspace{-6.49994pt}{\varSigma }_{DIC}$. Step 1 of the mCRE minimization thus yields after convergence:

36 $$\begin{aligned}  &   \left[ \mathbb {K}_{aa}({\textbf{p}}^{sol}) + \alpha .\frac{1}{N_{DIC}} (\varPi ^T \varSigma \hspace{-6.49994pt}{\varSigma }^{-1}_{DIC}\varPi )_{aa}\right] {\hat{{\textbf{U}}}}^{sol}_a \nonumber \\    &   \quad = \mathbb {K}_{aa}({\textbf{p}}^{sol}) {\hat{{\textbf{V}}}}^{sol}_a + \alpha .\frac{1}{N_{DIC}} (\varPi ^T \varSigma \hspace{-6.49994pt}{\varSigma }^{-1}_{DIC}\varPi )_{aa}{\hat{{\textbf{V}}}}^{sol}_a\nonumber \\  &   \qquad + \alpha .\frac{1}{N_{DIC}} (\varPi ^T \varSigma \hspace{-6.49994pt}{\varSigma }^{-1}_{DIC})_{ao}\varvec{\xi }\end{aligned}$$

so that

37 $$\begin{aligned} {\hat{{\textbf{U}}}}^{sol}_a  &   = {\hat{{\textbf{V}}}}^{sol}_a + \left[ \mathbb {K}_{aa}({\textbf{p}}^{sol}) + \alpha .\frac{1}{N_{DIC}} (\varPi ^T \varSigma \hspace{-6.49994pt}{\varSigma }^{-1}_{DIC}\varPi )_{aa}\right] ^{-1}\nonumber \\  &   \quad \alpha .\frac{1}{N_{DIC}} (\varPi ^T \varSigma \hspace{-6.49994pt}{\varSigma }^{-1}_{DIC})_{ao}\varvec{\xi }\end{aligned}$$

Consequently, the final value of the CRE term is:

38 $$\begin{aligned} \begin{aligned}&\mathcal {E}^{h^2}_{CRE}({\hat{{\textbf{U}}}}^{sol},{\hat{{\textbf{V}}}}^{sol};{\textbf{p}}^{sol}) \\&\quad = \frac{1}{2}({\hat{{\textbf{U}}}}^{sol}-{\hat{{\textbf{V}}}}^{sol})^T\mathbb {K}({\textbf{p}}^{sol})({\hat{{\textbf{U}}}}^{sol}-{\hat{{\textbf{V}}}}^{sol})\\  &\quad = \frac{1}{2}({\hat{{\textbf{U}}}}^{sol}_a-{\hat{{\textbf{V}}}}^{sol}_a)^T\mathbb {K}_{aa}({\textbf{p}}^{sol})({\hat{{\textbf{U}}}}^{sol}_a-{\hat{{\textbf{V}}}}^{sol}_a) \\&\quad = \frac{\alpha ^2}{2N^2_{DIC}}.\varvec{\xi }^T(\varPi ^T \varSigma \hspace{-6.49994pt}{\varSigma }^{-1}_{DIC})^T_{ao}{\overline{\mathbb {K}}}^{-T}_{aa}({\textbf{p}}^{sol},\alpha )\\  &\quad \mathbb {K}_{aa}({\textbf{p}}^{sol}){\overline{\mathbb {K}}}^{-1}_{aa}({\textbf{p}}^{sol},\alpha )(\varPi ^T \varSigma \hspace{-6.49994pt}{\varSigma }^{-1}_{DIC})_{ao}\varvec{\xi }\end{aligned} \end{aligned}$$

where we used the notation ${\overline{\mathbb {K}}}_{aa}({\textbf{p}}^{sol},\alpha ) = \mathbb {K}_{aa}({\textbf{p}}^{sol}) + \alpha .\frac{1}{N_{DIC}} (\varPi ^T \varSigma \hspace{-6.49994pt}{\varSigma }^{-1}_{DIC}\varPi )_{aa}$.

This value of the CRE term statistically tends to a scalar value denoted $\mathcal {E}^2_{ref}(\alpha )$. For a given $\alpha$, $\mathcal {E}^2_{ref}(\alpha )$ merely depends on measurement noise statistics and on the considered model; it can thus be computed in an *offline* phase, using for instance Monte-Carlo samplings. The value $\mathcal {E}^2_{ref}(\alpha )$ corresponds to the reference value of the CRE term, taking into account the impact of measurement uncertainties on optimal admissible fields through a model assumed to be perfect. It is therefore a value of choice for properly scaling the mCRE functional, and for comparison in adaptive modeling.

**Fig. 5:**
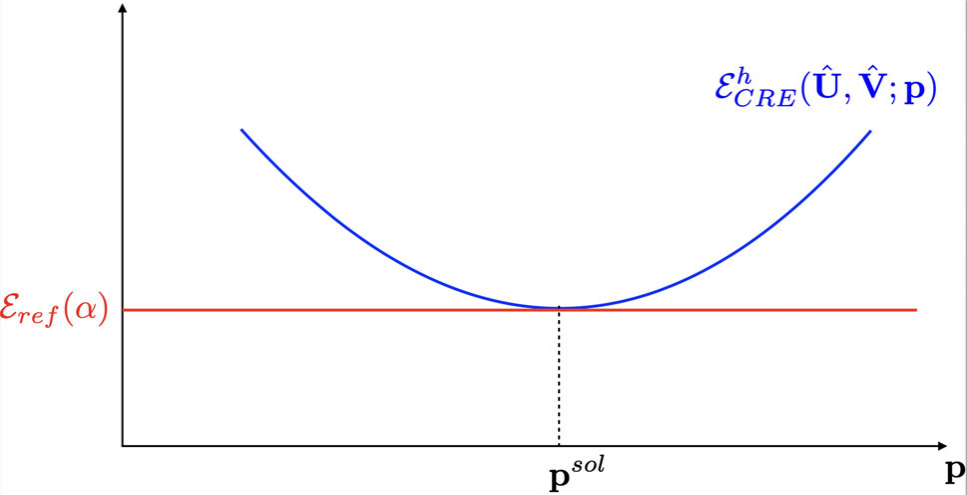
Optimal configuration which is searched at the end of the adaptive process, with similar values for the CRE term $\mathcal {E}^{h}_{CRE}$ (in blue) and the reference error $\mathcal {E}_{ref}$ (in red) at convergence of the mCRE minimization

Consequently, at the end of the identification process with ${\textbf{p}}={\textbf{p}}^{sol}$, the value of the CRE term $\mathcal {E}^{h^2}_{CRE}({\hat{{\textbf{U}}}}^{sol},{\hat{{\textbf{V}}}}^{sol};{\textbf{p}}^{sol})$ obtained in practice should be compared with the one given in (38) and corresponding to no mismatch between physics and modeling. This comparison naturally informs on the relevance of the chosen linear elastic mathematical model:

- when the model is compatible with (noisy) observations, we should get $\mathcal {E}^{h}_{CRE}({\hat{{\textbf{U}}}}^{sol},{\hat{{\textbf{V}}}}^{sol};{\textbf{p}}^{sol})/\mathcal {E}_{ref}(\alpha ) \approx 1$ (=1 on average); this is the configuration shown in Fig. 5;
- when $\mathcal {E}^{h}_{CRE}({\hat{{\textbf{U}}}}^{sol},{\hat{{\textbf{V}}}}^{sol};{\textbf{p}}^{sol})/\mathcal {E}_{ref}(\alpha ) \gg 1$, this indicates bias in the employed mathematical model. This model is then too poor to represent the partial physical reality seen from observations;
- when $\mathcal {E}^{h}_{CRE}({\hat{{\textbf{U}}}}^{sol},{\hat{{\textbf{V}}}}^{sol};{\textbf{p}}^{sol})/\mathcal {E}_{ref}(\alpha ) \ll 1$, the employed mathematical model appears to be too rich with regards to available experimental information.

We remind that the value of $\alpha$ is in practice calibrated in a systematic manner by satisfying the Morozov principle, that is:

39 $$\begin{aligned} \frac{1}{N_{DIC}}(\varPi {\hat{{\textbf{U}}}}^{sol}-{\textbf{U}}_{DIC})^T\varSigma \hspace{-6.49994pt}{\varSigma }_{DIC}^{-1}(\varPi {\hat{{\textbf{U}}}}^{sol}-{\textbf{U}}_{DIC}) \approx 1 \end{aligned}$$

which indicates that DIC data are approached up to noise level.

The value $\mathcal {E}^{h}_{CRE}({\hat{{\textbf{U}}}}^{sol},{\hat{{\textbf{V}}}}^{sol};{\textbf{p}}^{sol}) = \frac{1}{2}({\hat{{\textbf{U}}}}^{sol}-{\hat{{\textbf{V}}}}^{sol})\mathbb {K}({\textbf{p}}^{sol})({\hat{{\textbf{U}}}}^{sol}-{\hat{{\textbf{V}}}}^{sol})$ of the CRE term in the discretized mCRE functional can thus be interpreted, at convergence of the identification algorithm, as a modeling error indicator denoted $\eta _{mod}$. Computed from optimal admissible fields, it informs on the quality of the identified (here linear elasticity) model in view of noisy measurements, when compared with the reference value $\mathcal {E}_{ref}(\alpha )$.

### Indicator on the discretization error

In addition to the material law chosen, bias in the identified computational model may also come from discretization error, that is the approximation made when using a FE mesh $\mathcal {T}_h$ to represent admissible fields and the state of the system. This part of the error can not be captured by $\eta _{mod}$ as this indicator is derived from a discretized version of the mCRE functional, and it thus refers to a model which is already discretized in space. To assess the discretization error part, and therefore evaluate the strict quality of the mesh, one needs to refer to a continuous reference model. Consequently, a fully equilibrated stress field denoted  should be computed, similarly to tools using the CRE concept which have been developed for more than 40 years to perform model verification and mesh adaptivity in the FE context (see [60, 62] for an overview). In other words, this field  should satisfy:40In practice, such a fully equilibrated stress field can be obtained from a direct post-processing of the stress field associated with ${\hat{{\textbf{V}}}}^{sol}$ at hand, that is  with $\mathbb {N}$ the matrix of FE shape functions. Indeed, due to admissibility constraints enforced in the mCRE formulation,  satisfies equilibrium in the FE sense, that is:

41 $$\begin{aligned} \delta {\textbf{V}}^T\left( \mathbb {K}({\textbf{p}}){\hat{{\textbf{V}}}}^{sol}-{\textbf{F}}\right) =0 \quad \forall \delta {\textbf{V}}\in \mathcal {U}_{ad,S}^{h,0} \end{aligned}$$

The employed *a posteriori* recovery procedure corresponds to the so-called hybrid-flux technique initially developed for model verification in [55], with several variants proposed over the years, e.g. in [36, 61, 82, 83]. Additional details on this technique are given in the next section.

#### Remark 3

Alternative techniques can be employed to get a fully equilibrated stress field in the (m)CRE-based material identification framework. For instance, Airy functions are used in [40, 63]. In [37, 72], a global finite-dimensional optimization problem is solved by considering a regular simulation grid; unknowns are then equilibrated polynomial tractions over element edges, after defining a direct link between these tractions and the inside local stress field. Nevertheless, such techniques do not offer as much flexibility (e.g., in terms of geometry of the domain $\varOmega$) as the one we propose.

Once  is obtained, an error indicator $\eta _{tot}$ accounting for all (i.e., both model class and discretization) error sources can naturally be computed with the CRE term. It is defined as42Eventually, from orthogonality properties, an error indicator $\eta _{dis}$ on the discretization error alone can be designed as:

43 $$\begin{aligned} \eta ^2_{dis} \equiv \eta ^2_{tot} - \eta ^2_{mod} \end{aligned}$$

While $\eta _{mod}$ informs on the error due to the mathematical model alone, we intend that $\eta _{dis}$ informs on the discretization error alone.

#### Remark 4

When generating synthetic measurement data using a FE mesh, as done for some of the numerical experiments performed in Sect. 4, one should consider a refined mesh compared to the one used for the computation of optimal admissible fields in the mCRE-based identification. If the same mesh is employed for both, there is no discretization error with respect to measurements, so that the indicator $\eta _{dis}$ on discretization error would be close to 0.

#### Remark 5

In the general case of nonlinear dissipative material laws, as discussed in Sect. 2.4, the construction of a continuous admissible solution (${\hat{{\textbf{e}}}}^{sol}$,${\hat{{\textbf{s}}}}^{sol}$) can again be conducted from the discrete optimal admissible solution (${\hat{{\textbf{e}}}}^{sol}_h$,${\hat{{\textbf{s}}}}^{sol}_h$) represented on a mesh $\mathcal {T}_h$ and known at discrete time points $t_n$. This discrete solution is first extended across the whole time-space domain, in order to satisfy kinematic constraints and weak FE equilibrium at any time $t \in [0,T]$, then the hybrid-flux equilibration technique is employed to compute (${\hat{{\textbf{e}}}}^{sol}$,${\hat{{\textbf{s}}}}^{sol}$). All details on this construction can be found in [57, 60].

### Computation of equilibrated fields

Here, we briefly summarize the main ingredients of the hybrid-flux equilibration technique which permits to recover a fully-equilibrated stress field from the post-processing of a stress field which is equilibrated in a weaker FE sense. The technique is made of two steps:

1. construction of polynomial tractions ${\hat{{\textbf{f}}}}_{K|\varGamma }$, balanced with the external loading (${\textbf{f}}^v_d$, ${\textbf{f}}^s_d$), on edges $\varGamma$ of each element *K* of the mesh $\mathcal {T}_h$. They should satisfy ${\hat{{\textbf{f}}}}_{K|\varGamma }={\textbf{f}}^s_d$ if $\varGamma \subset \partial _2 \varOmega$, as well as equilibrium at the element level:
  44 $$\begin{aligned} \int _K {\textbf{f}}^v_d \cdot {\textbf{u}}^{*}_R + \int _{\partial K}{\hat{{\textbf{f}}}}_K \cdot {\textbf{u}}^{*}_R =0 \quad \forall {\textbf{u}}^{*}_R \in \varvec{\mathcal {U}}_R(K) \end{aligned}$$
  where $\varvec{\mathcal {U}}_R(K)$ denotes the space of rigid body motions on *K*.
2. in each element *K*, construction of a stress field  that satisfies equilibrium: 45 The associated local and independent problems are in practice solved with a quasi-explicit technique and polynomial basis, or with a dual approach.

**Fig. 6:**
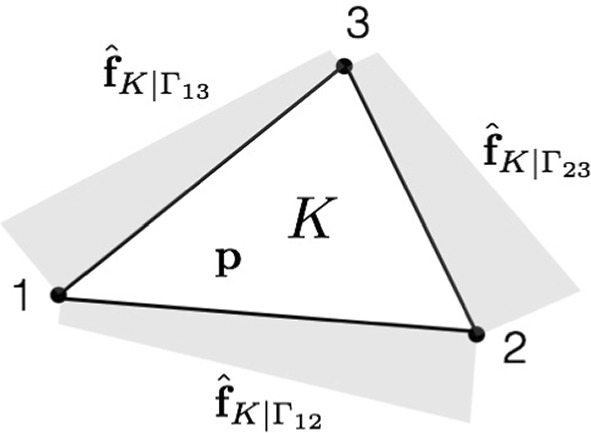
Configuration at the level of a 3-node triangle element, with linear tractions ${\hat{{\textbf{f}}}}_{K|\varGamma }$ defined on element edges

Here again, the PGD model reduction technique with offline/online strategy can be advantageously used. Indeed, due to the multi-query context induced by the hybrid-flux technique, local problems (45) need to be solved for various material parameter ${\textbf{p}}$ values, element geometrical shapes, and boundary conditions (see Fig. 6). After parametrization of local problems, a parametrized PGD solution is computed in the offline phase then evaluated for any configuration in the online phase with much lower computational cost. Full details on this PGD procedure in the context of stress field equilibration can be found in [1, 16].

**Fig. 7:**
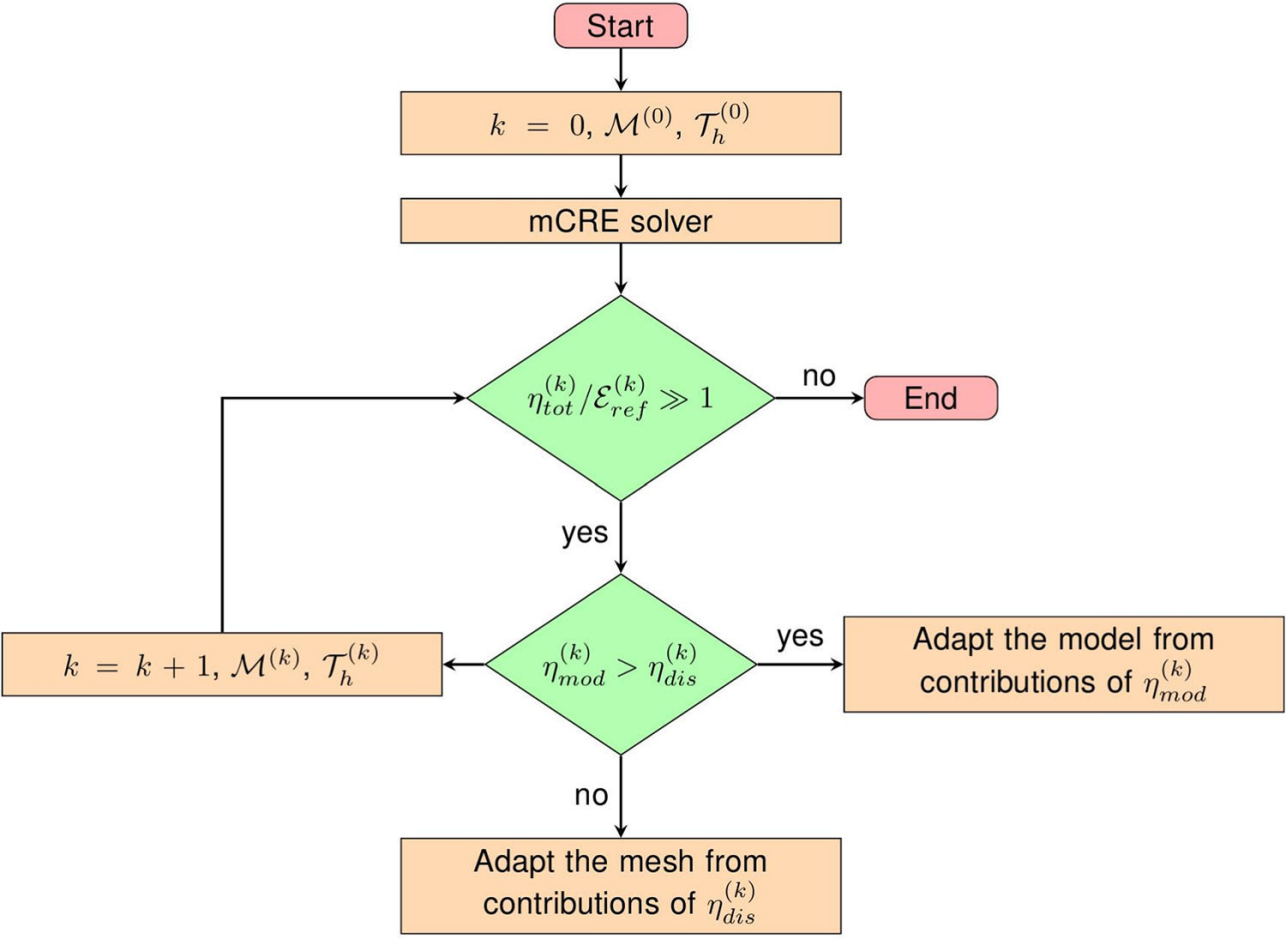
Scheme of the adaptive process

### Adaptive strategy

The previously defined error estimates $(\eta _{tot},\eta _{mod},\eta _{dis})$ can naturally be used, at the end of the identification process, as quantitative criteria (i) to define whether or not the computational model is appropriate with respect to observations, and (ii) to potentially drive the selection of a more convenient model among a hierarchy of models with increasing complexity (multi-fidelity approach). By comparing with the reference error $\mathcal {E}_{ref}$, these estimates are thus involved in the design of an adaptive procedure in which higher-fidelity models in terms of model class and mesh size should be progressively inserted when dictated by data.

In practice, starting from an initial (coarse) computational model with model class $\mathcal {M}^{(0)}$ and mesh $\mathcal {T}_h^{(0)}$, adaptivity is performed in a greedy manner at each iteration *k*. When $\eta ^{(k)}_{tot} \gg \mathcal {E}^{(k)}_{ref}$, indicating that the overall computational model is not consistent with experimental information, adaptivity is conducted by comparing relative values of error indicators $\eta ^{(k)}_{mod}$ and $\eta ^{(k)}_{dis}$, as well as their local spatial contributions, to perform local adaptation. Also, results obtained from lower-fidelity models i.e., optimal admissible fields and identified parameter values, are reused in order to regularize the identification of higher-fidelity models. A scheme of the adaptive algorithm is given in Fig. 7.

**Fig. 8:**
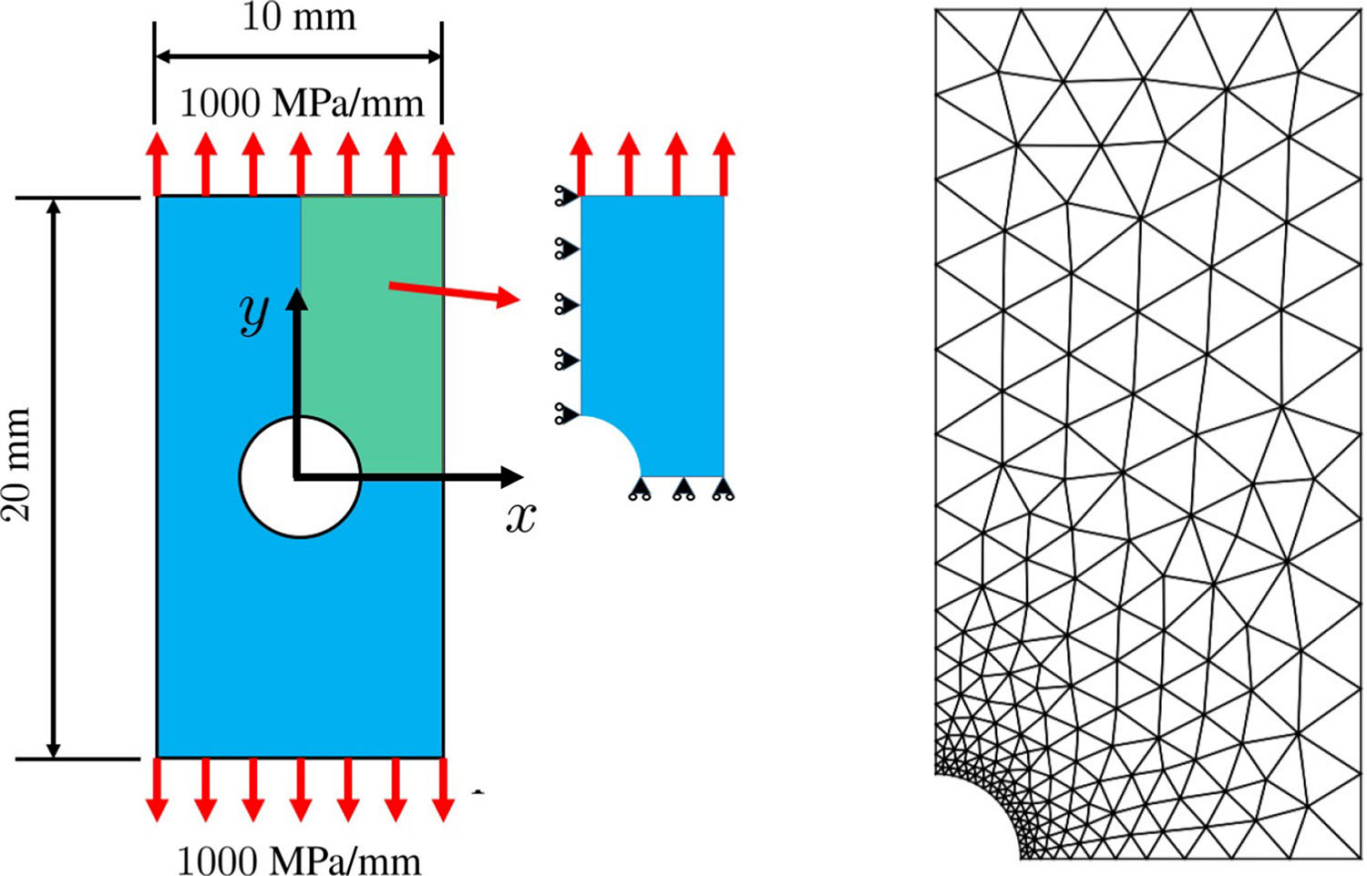
Model problem with tensile test on a plate with hole (left), and associated mesh (right)

At the end of the adaptive process, the algorithm should tend to an optimized identified model that is consistent with data, yielding savings in modeling complexity without sacrificing accuracy. We highlight that optimality has to be understood with the objective of identification; adapted model and mesh, dedicated to parameter identification and implicitly considering sensitivity analysis and experimental information, are strongly coupled with the amount and richness of measurements. They differ from adapted model and mesh that should be used for accurate model-based prediction in a direct problem.

#### Remark 6

When adapting the model class, the selection of a new higher-fidelity model is here performed empirically among a given list models. It may be possible to design a more elaborate procedure in which optimal admissible fields at hand (which remain admissible for any constitutive equation) are tested with other CRE functionals to assess sensitivity on the CRE value and give insight on the appropriate direction to be followed for searching a better model. This way, sensitivity analysis is performed in a cheap manner over the *a priori* chosen manifold of material models i.e., catalogue of constitutive classes. Nevertheless, at the end of the process, optimal admissible fields should still be computed to ultimately quantify the quality of the new chosen model. Alternatively, a parsimonious and more automatic selection could be envisioned by using sparse regression over the (possibly large) number of candidate material models, as performed in the EUCLID method [34].

#### Remark 7

In practice, a change in model class is here performed over the whole domain $\varOmega$, but coupling between concurrent models (e.g., with local-global or multi-grid approaches) could also be envisioned in order to permit local changes. This would be a relevant strategy for keeping coarse model and mesh in regions which are not sensitive to identified parameters.

#### Remark 8

We define here an adaptive process which is driven by the measurement noise level. We indicate that the reverse procedure could also be possible, that is with given reference model and mesh, we could define the richness of experimental information which is consistent to use. In the case of full-field measurements, this would come down to selecting a consistent image resolution (e.g. compressing data images with SVD) or a suitable correlation mesh (e.g. to get iso-measurement noise) within a multiscale storage of images. This constitutes a pruning process, implicitly based on measurement reliability, that enables saving in the amount of data [75, 81].

## Numerical results

### Application 1: identification of homogeneous elastic properties of an orthotropic plate

As a first illustration, we consider the identification of elastic properties of a plate with a hole (Fig. 8), from a uniaxial or biaxial tensile test. Using symmetry properties, only one quarter of the plate is conserved. An example of locally refined simulation mesh made of T3 elements, considered later when dealing with the assessment of discretization error, is also shown in Fig. 8. We use here synthetic displacement measurements obtained from a reference homogeneous orthotropic elastic material behavior with coefficients $E^0_{x}=130$ GPa, $E^0_{y}=10$ GPa, $G^0_{xy}=5$ GPa, and $\nu ^0_{xy}=0.35$. From kinematic data generated from this reference model, an additive noise (uncorrelated Gaussian white perturbation) is added.

#### Model selection

**Fig. 9:**
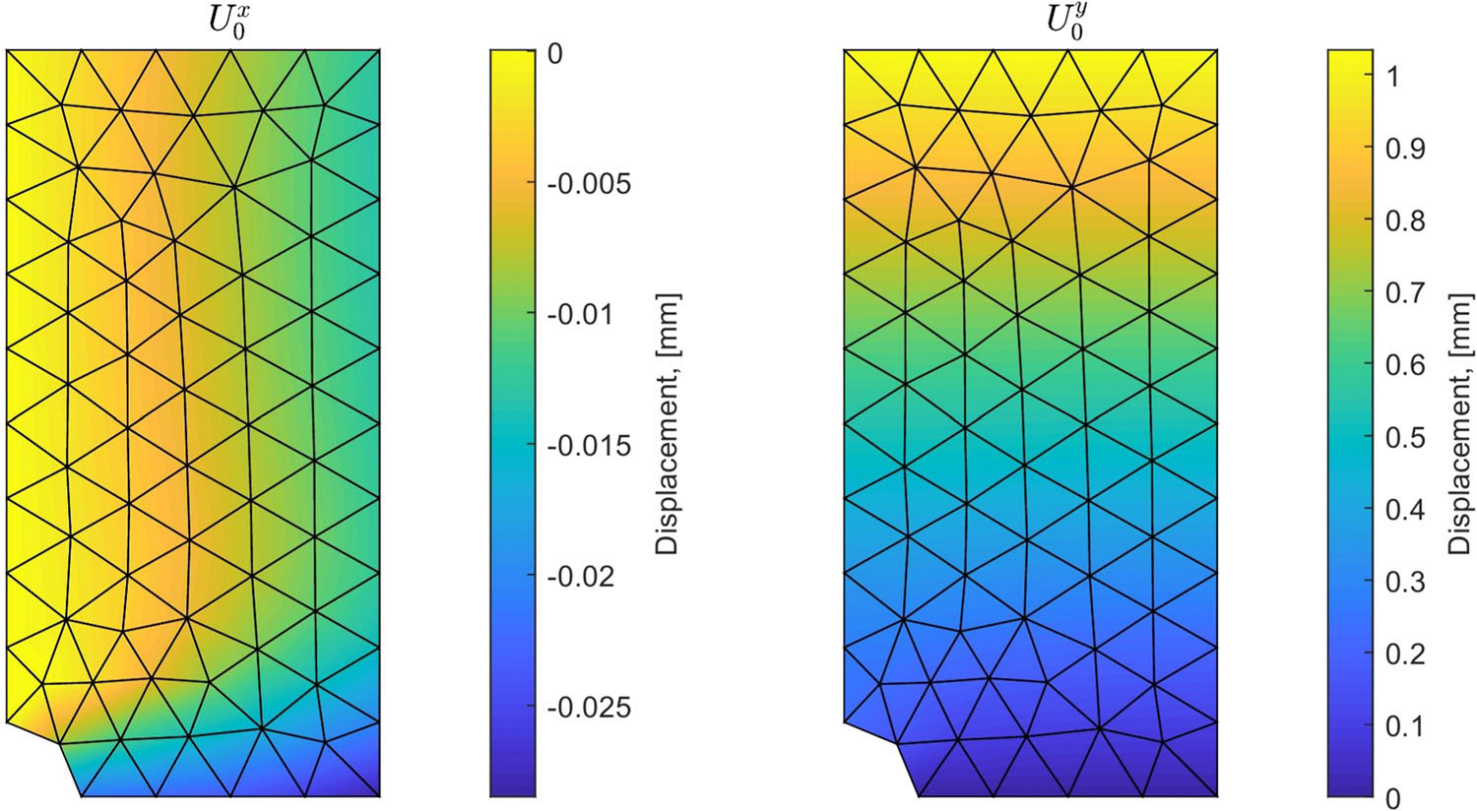
Reference solution used to obtain synthetic data for identification

**Fig. 10:**
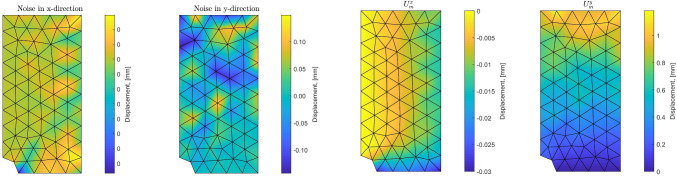
Noise distribution (left) and resulting noisy displacement field used as synthetic measurements (right), for a 10% noise level

First, we test the ability of the mCRE-based identification procedure and error assessment to drive the adaptive selection of an appropriate model class; discretization error is not considered at this stage. In this context, the same regular coarse mesh is used for data generation and mCRE computations. Considering first a uniaxial loading of 1000 Mpa/mm in the *y* direction, the reference solution ${\textbf{U}}_0$ computed from the reference set of material properties is shown in Fig. 9. An additive 10% noise is then added, leading to the displacement field shown in Fig. 10 and used as synthetic data.

**Fig. 11:**
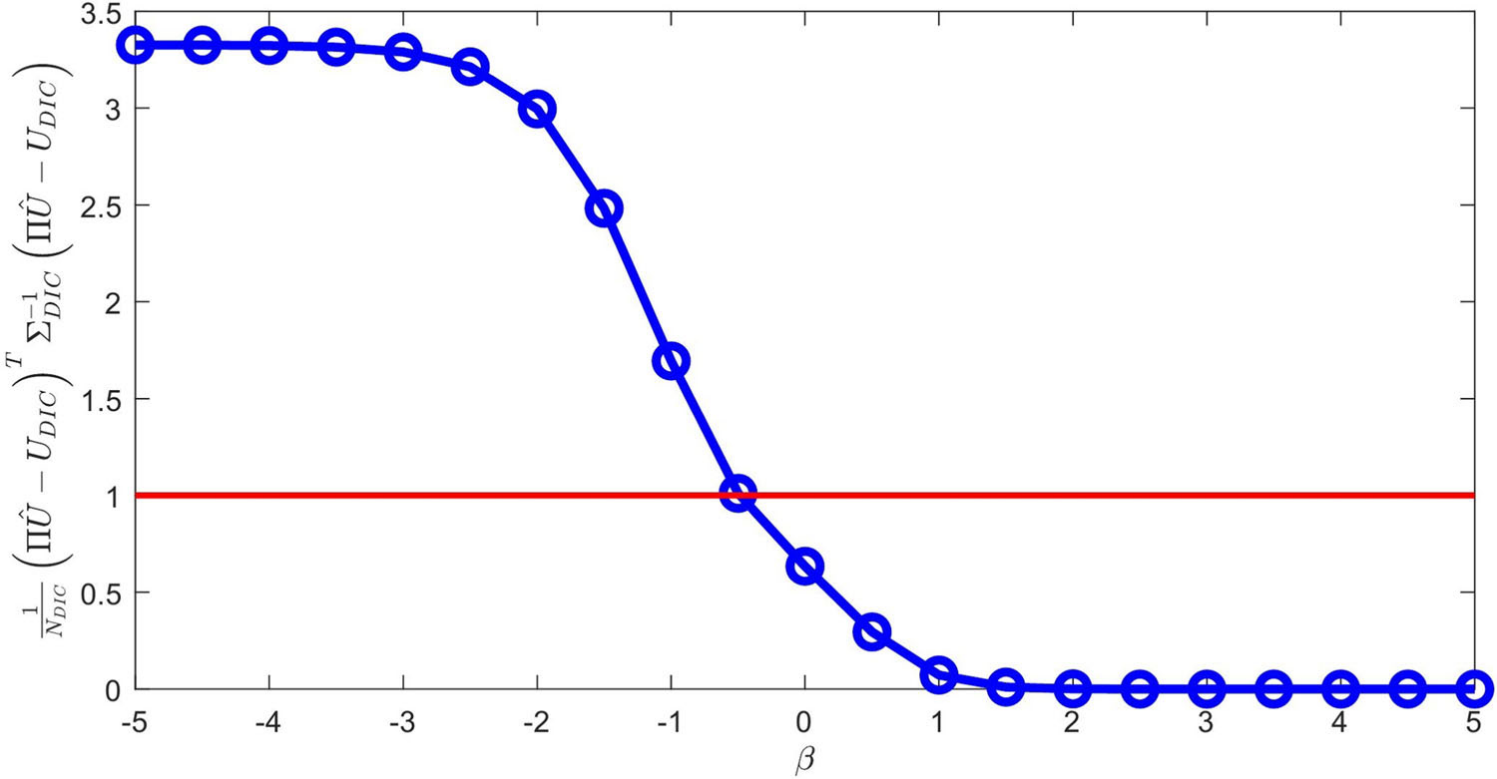
Evolution of the term on discrepancy with measurements as a function of $\beta$, for an isotropic elastic computational model

Starting from *a priori* knowledge on homogeneity and on the value of the Poisson ratio alone ($\nu = 0.35$), an isotropic elasticity model class $\mathcal {M}^{(0)}$ is initially used to identify the associated Young modulus *E*. The weighting factor $\alpha$ of the mCRE is defined as $\alpha = 10^\beta . {\textbf{U}}_0^T\mathbb {K}_0{\textbf{U}}_0$ where ${\textbf{U}}_0$ and $\mathbb {K}_0$ are displacement field and stiffness matrix associated with the reference solution. The coefficient $\beta$ is tuned in the range $[-5,5]$, and the curve associated with the Morozov principle is represented in Fig. 11. We observe that when $\beta$ is too small, there is loss of information contained in the measurements and over-smoothing of the solution, resulting to values of the model-data discrepancy term larger than 1. Nevertheless, the curve does not go too high for small $\beta$, as admissible fields which are compared to measurements are already constructed from both model and measurements, so that model correction from measurement data is already performed. The curve indicates that the optimal weight value is around $\beta =-0.5$. For this value, the identified value is $E \approx E_{y,0} = 10$ GPa and the error estimate $\eta _{tot}$ indicates the computational model is quite compatible with data ($\eta _{tot}^{(0)}/\mathcal {E}^{(0)}_{ref}=3.92$), as such data obtained from a uniaxial tensile test is not rich enough to detect the mismatch.

**Fig. 12:**
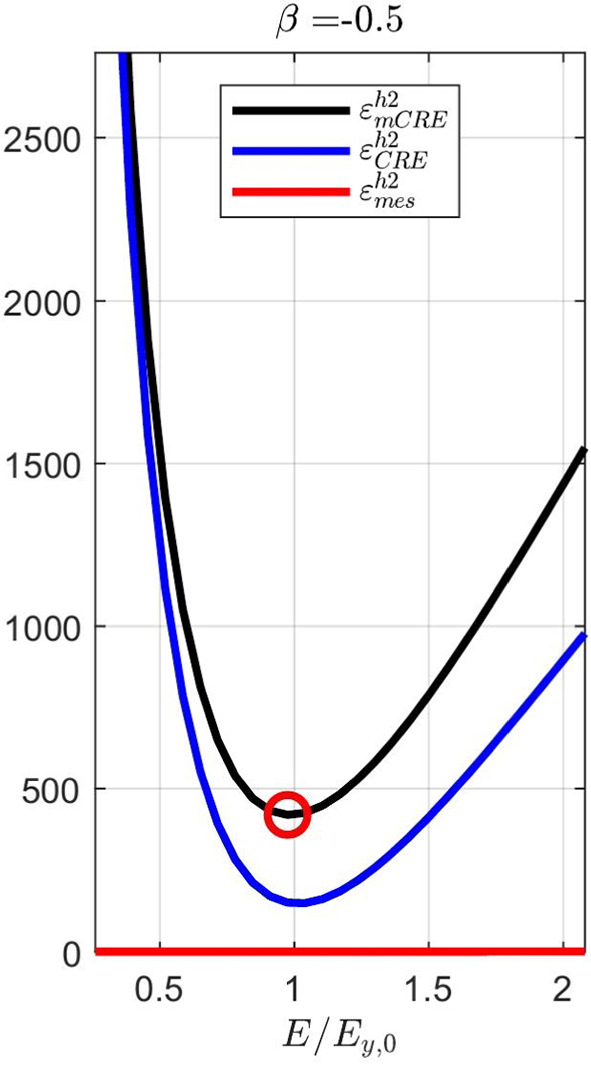
Evolutions of the mCRE functional and its two components with $\beta =-0.5$ for a biaxial tensile test

Considering now a biaxial tensile test, with added loading of 200 MPa/mm in the *x* direction, the evolution of the mCRE cost function and its two components is displayed in Fig. 12. We still observe that the identified value is $E \approx E_{y,0} = 10$ GPa, and that the cost function has good convexity properties.

**Fig. 13:**
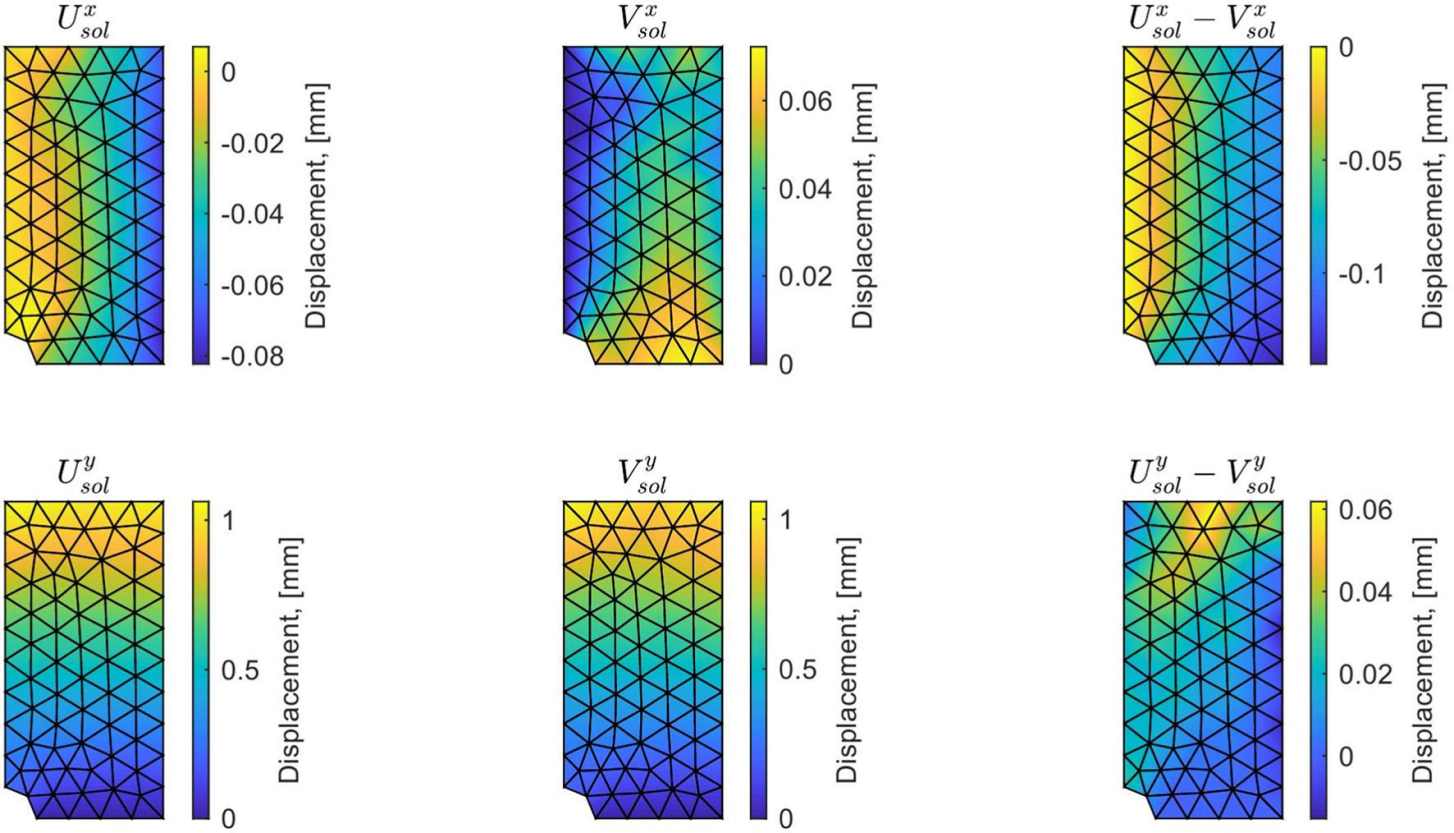
Spatial representation of admissible fields ${\hat{{\textbf{u}}}}^{sol}$ (left), ${\hat{{\textbf{v}}}}^{sol}$ (center), and their difference (right) at iteration 1 of the greedy algorithm

We represent in Fig. 13 the optimal admissible fields ${\hat{{\textbf{u}}}}^{sol}$ and ${\hat{{\textbf{v}}}}^{sol}$ (and their difference) obtained at the end of the identification process. The difference is quite large, which informs on bias in the computational model.

Computing the error estimates, we get with this initial computational model (isotropic elasticity material law and coarse mesh) the following normalized values:

46 $$\begin{aligned} \eta ^{(0)}_{tot}/\mathcal {E}^{(0)}_{ref}  &   = 47.2 \quad ; \quad \eta ^{(0)}_{mod}/\mathcal {E}^{(0)}_{ref} = 46.8 \quad ; \nonumber \\  &   \quad \eta ^{(0)}_{dis}/\mathcal {E}^{(0)}_{ref} = 6.1 \end{aligned}$$

The indicator $\eta _{dis}$ is much lower than the indicator $\eta _{mod}$, even though it is not exactly 0 as it merely assesses the exact discretization error value. Consequently, from the greedy algorithm, the model class is then changed into an orthotropic elasticity model, with a larger set of associated parameters to be identified, but with a mesh size kept unchanged.

**Fig. 14:**
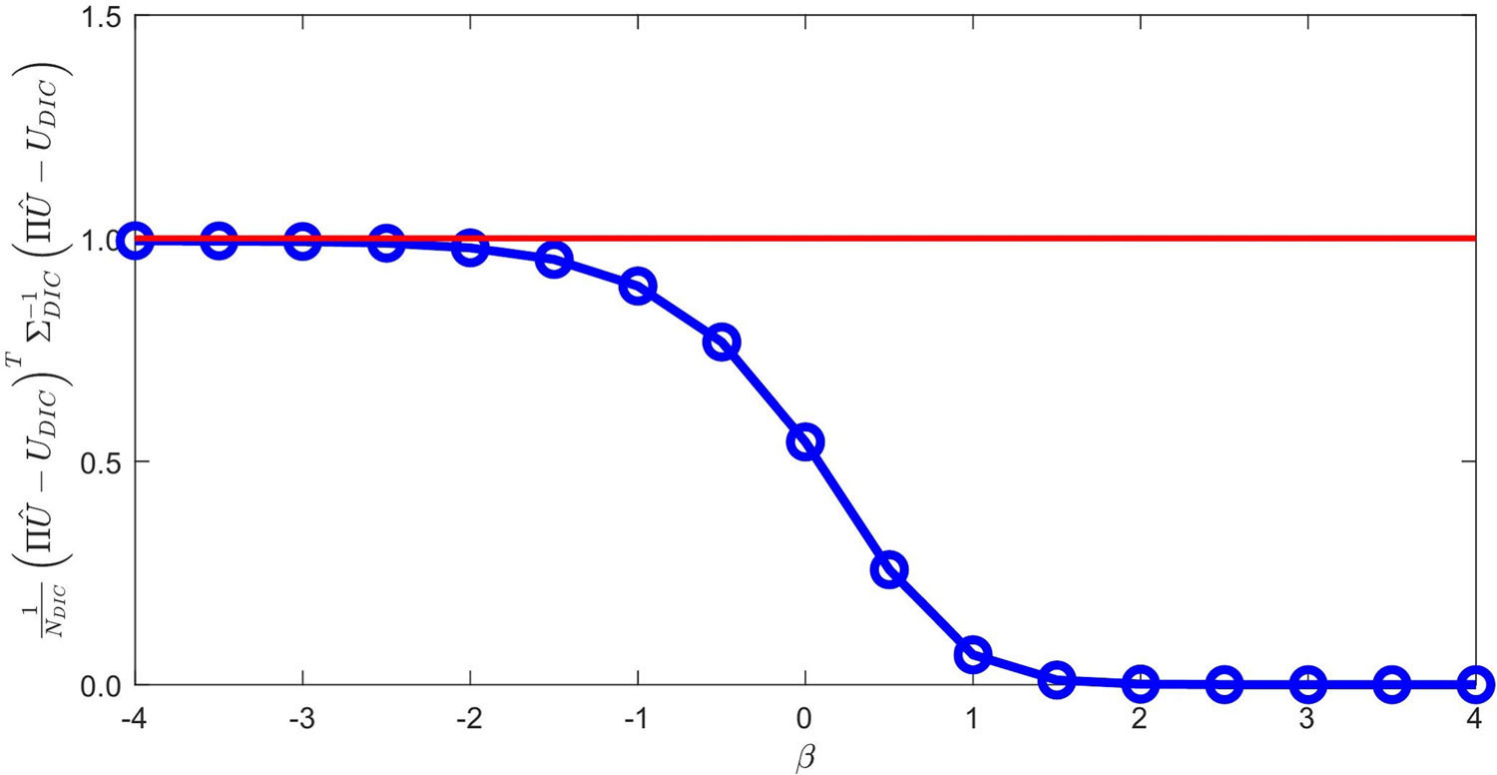
Evolution of the term on discrepancy with measurements as a function of $\beta$, for an orthotropic elastic computational model

In the second identification process, the coefficient $\beta$ is now tuned in the range $[-4,4]$, and the curve associated with the Morozov principle is represented in Fig. 14. This time, we observe that for small $\beta$, and as the model is here compatible with experimental information, the weighted term on model-data discrepancy stagnates to the desired target which is the value 1. Conversely, choosing a too large $\beta$ makes this term go to 0, which is undesirable in the case of noisy data. In this specific example, we select the value of $\beta$ that satisfies the criterion on Morozov principle (with measurement data fit up to noise level) while not over-smoothing too much the solution from the model. The curve thus indicates here to choose $\beta =-1.5$.

**Fig. 15:**
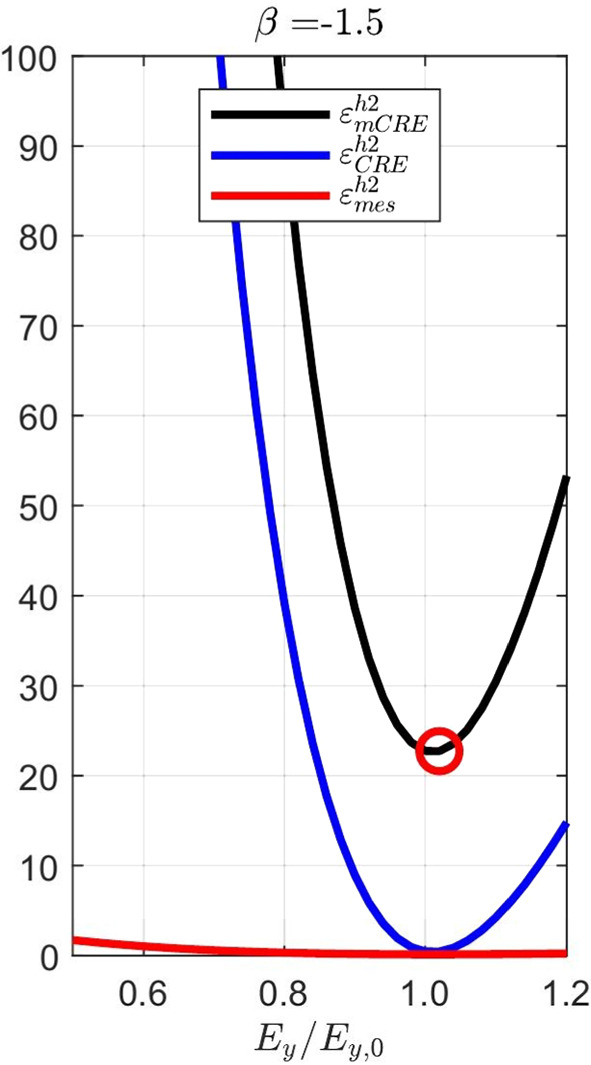
Evolutions of the mCRE functional and its two components with $\beta =-1.5$

**Fig. 16:**
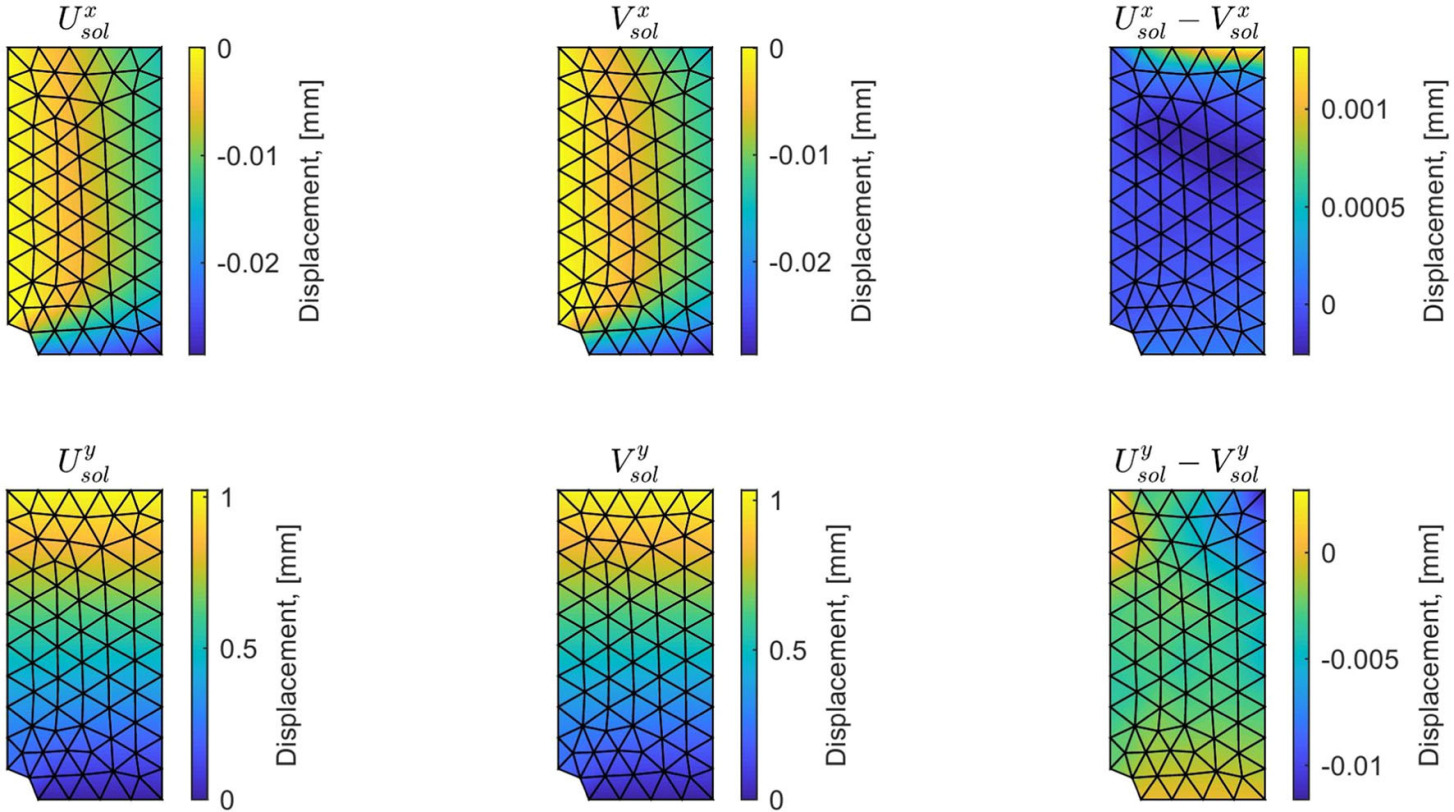
Spatial representation of admissible fields ${\hat{{\textbf{u}}}}^{sol}$ (left), ${\hat{{\textbf{v}}}}^{sol}$ (center), and their difference (right) at iteration 2 of the greedy algorithm

For this optimal value of $\alpha$, the evolution of the mCRE functional and its two components is represented with respect to parameter $E_y$ (other parameters being set to their converged value) in Fig. 15. We observe that the value of $E_y$ is correctly identified ($E_y \approx E_{y,0} = 10$ GPa). The optimal admissible fields ${\hat{{\textbf{u}}}}^{sol}$ and ${\hat{{\textbf{v}}}}^{sol}$ and their difference obtained at the end of the identification process are represented in Fig. 16. The difference is now negligibly small as the model is correctly chosen, and we get $\eta ^{(1)}_{mod}/\mathcal {E}^{(1)}_{ref}=1.9$ so that the adaptive algorithm can be stopped.

#### Mesh selection

We now consider synthetic data obtained from the same reference model but with the refined mesh given in Fig. 8. An additive 10% noise is again added.

**Fig. 17:**
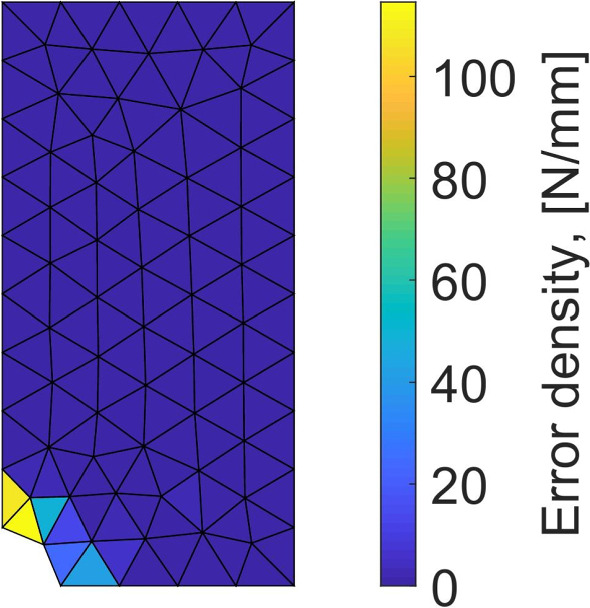
Density of local discretization error

Performing the mCRE-based identification process with the linear orthotropic elasticity model class selected previously, we now obtain:

47 $$\begin{aligned}  &   \eta _{tot}/\mathcal {E}_{ref} = 15.2 \quad ; \quad \eta _{mod}/\mathcal {E}_{ref} = 2.1 \quad ; \nonumber \\  &   \quad \eta _{dis}/\mathcal {E}_{ref} = 15.1 \end{aligned}$$

which indicates that the computation mesh should be refined while keeping the model class unchanged. The discretization error density is shown in Fig. 17.

**Fig. 18:**
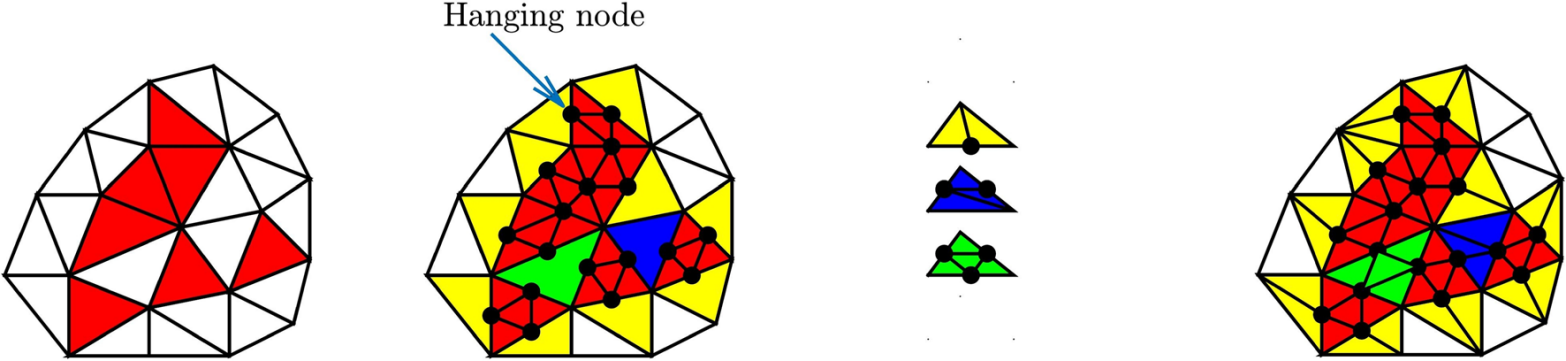
Illustration of the mesh refinement process

Mesh adaptation is performed using subdivision of existing elements (with management of hanging nodes) until error indicators have converged to acceptable values. The reason of preferring a subdivision method for mesh refinement instead of local remeshing comes from the fact that the mCRE method is performed at each iteration, with simplified projection between consecutive meshes. The process of subdivision is illustrated in Fig. 18. Red elements are of largest discretization error density and are chosen to be subdivided. To perform subdivision, new nodes are created at the midpoints of element edges. With these newly created nodes, each of the red elements is subdivided into 4 smaller ones. However, after subdivision, hanging nodes appear and to manage them, the associated adjacent elements are also subdivided as follows: (i) elements with 1 hanging node (yellow ones) are divided into 2 smaller ones; (ii) elements with 2 hanging nodes (blue ones) are divided into 3 smaller ones; (iii) elements with 3 hanging nodes (green ones) are divided into 4 smaller ones. The resulting mesh (on the right) no longer has hanging node.

**Fig. 19:**
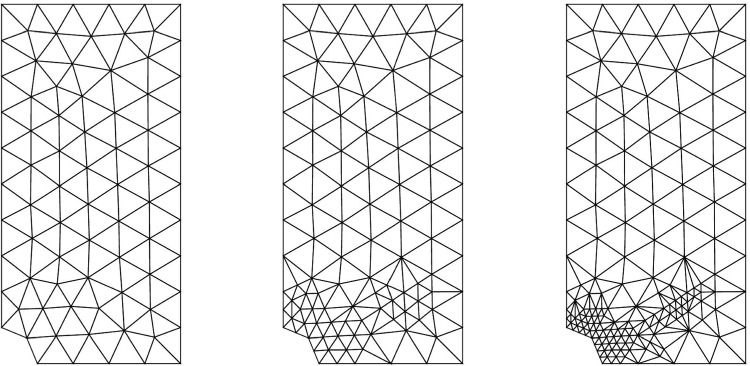
Meshes obtained along the adaptive algorithm, at iteration 1 (left), iteration 2 (center), and iteration 3 (right)

**Fig. 20:**
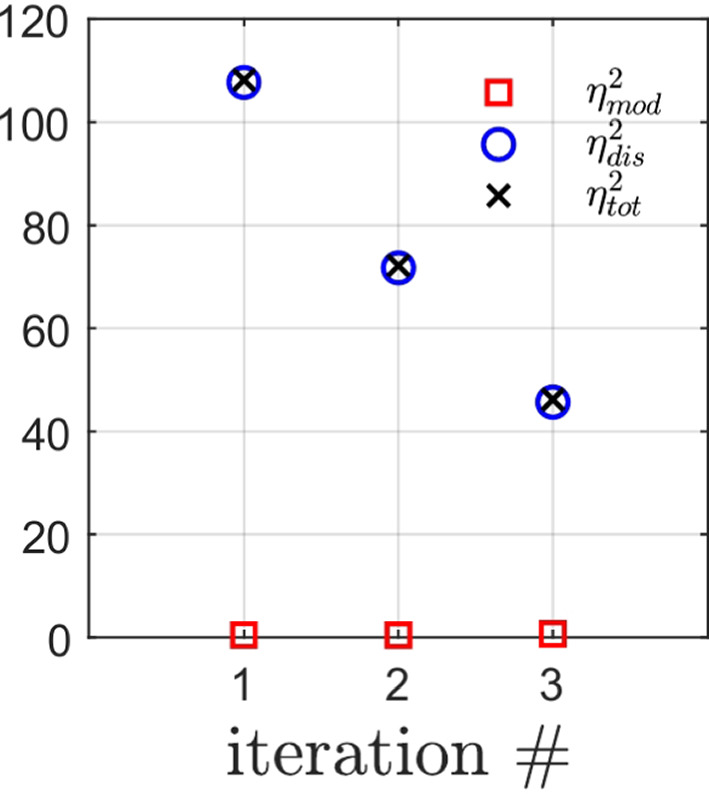
Error indicators at each iteration of the adaptive algorithm

**Fig. 21:**
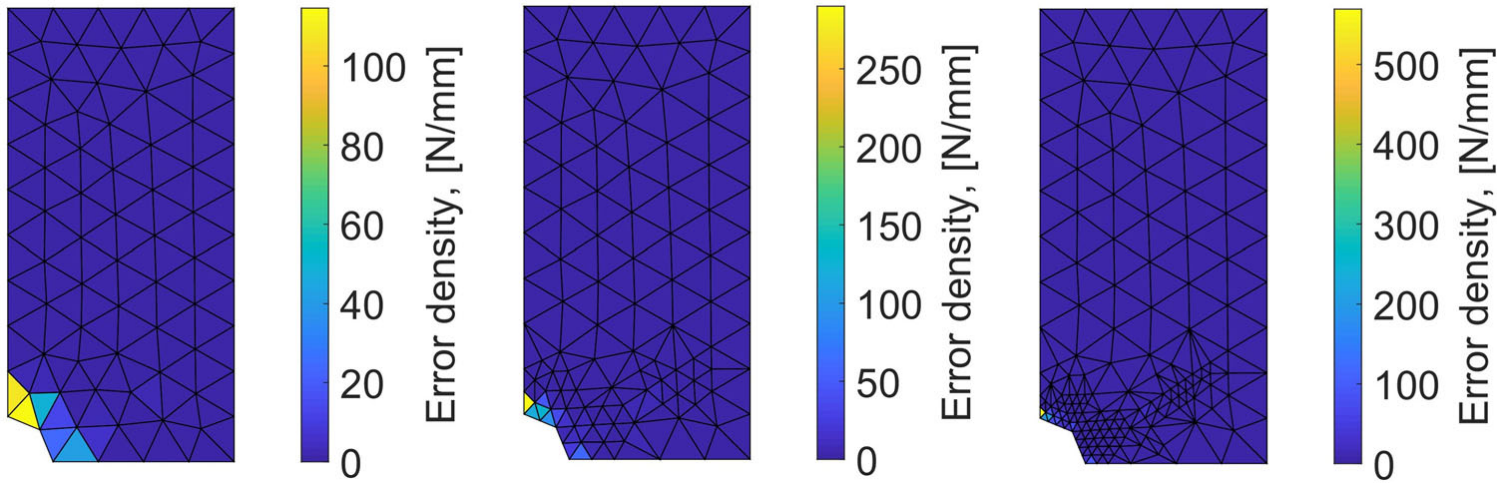
Discretization error density at iterations 1 (left), 2 (center), and 3 (right)

Using the greedy adaptive algorithm, the mesh is iteratively refined. The obtained sequence of meshes is shown in Fig. 19; it is consistent with the mechanical problem, and the algorithm stops at iteration 3. We highlight that during this adaptive process, the evolution of the term on discrepancy with measurements as a function of $\beta$ remains almost the same, so that the optimal $\beta$ value is kept unchanged with $\beta =-1.5$. The values of error indicators obtained along the adaptive process are shown in Fig. 20. We observe that the model class error indicator $\eta _{mod}$ is small for all iterations due to the correct initial choice of the mathematical model, while the discretization error indicator $\eta _{dis}$ gradually reduces with mesh refinements. Also, the spatial distribution of the discretization error density (used to perform mesh refinement) is shown in Fig. 21. It can be observed that, for regions with no refinement, the density remains small (nearly zero), while in the refinement regions the error density increases due to the concentration of the error in smaller and smaller elements.

### Application 2: identification of elastoplastic properties of a specimen with notches

**Fig. 22:**
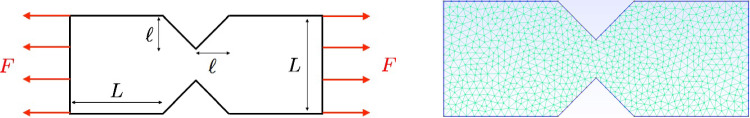
Model problem with tensile test on a specimen with notches (left), and associated mesh (right)

We now consider a tensile test on a specimen with notches, as represented in Fig. 22. A uniaxial loading with evolving magnitude *F* is applied on left and right edges; other sides are free.

Synthetic measurement data are obtained using a reference solution generated from the Prandtl-Reuss plastic model with kinematic hardening (see below). The associated reference values are $E=200$ GPa, $\nu =0.3$, $R_0=160$ MPa, $A=0.63$ MPa$^{-1}$, and $C=48$ GPa. A 5% additive Gaussian white noise is added.

**Fig. 23:**
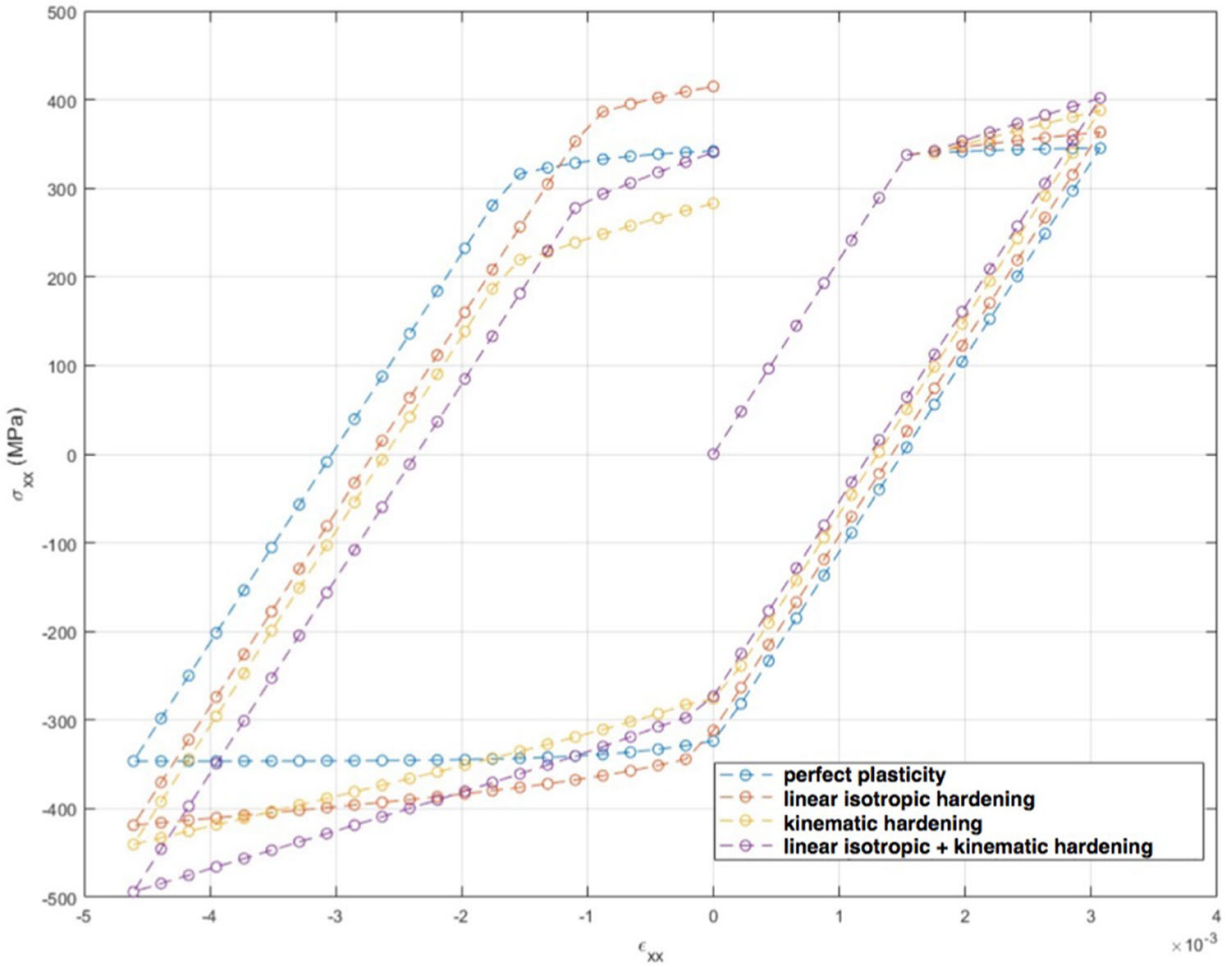
Relative evolution of stress-strain components ($\sigma _{xx}$, $\epsilon _{xx}$) during the loading history

**Fig. 24:**
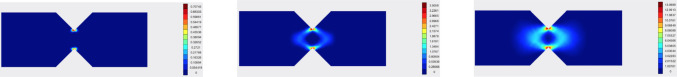
Maps of the cumulative plastic strain field at three loading increments

The considered loading is pseudo-cyclic, with successive loading-unloading-reloading steps such that *F* goes higher than the threshold loading $F^*$ where plasticity is effectively observed in the FEM simulation (theoretically, it appears immediately due to the singularity). The associated evolution curve of longitudinal stress-strain components is shown in Fig. 23, while the progressive apparition of cumulative plastic strain is shown in Fig. 24.

In the following, we perform identification with the mCRE method using concurrent mathematical models; the discretization error is here discarded by initially using a fine mesh (see Fig. 22). The only *a priori* information which is introduced in the model class is that material properties are homogeneous. We also assume that the Poisson ratio is known.

#### mCRE formulation with a Prandtl-Reuss plastic model

In this section, we detail the thermodynamic potentials and the writing of the mCRE functional for several versions of Prandtl-Reuss plastic models which will be used along the adaptive process.

We first start with the *Prandtl-Reuss model with isotropic hardening*. For this model, we have:48with  the cumulative inelastic strain ($\Vert \bullet \Vert = (\bullet : \bullet )^{1/2}$) and *R* the associated thermodynamic force that corresponds to the isotropic hardening variable on additional yield stress. The associated free energy potential reads  with *g* a function that characterizes the hardening law (e.g., $g(p)=\frac{1}{2}k p^2$ for linear hardening, with *k* a strictly positive material parameter). We thus obtain the following state laws:49and the dual potential reads:  with $g^*$ the Legendre-Fenchel transform of function *g*.

As regards the dissipation potential , it is the indicator function of the elasticity domain , that is:50with  for linear hardening and  for power hardening,  the deviatoric part of the stress tensor, and $R_0\ge 0$ the yield stress.

Introducing the convex set  with associated indicator function $\varPsi _{C_e}$, the dual dissipation potential reads (for linear hardening):51

##### Remark 9

In the viscoplastic case (with linear hardening), the dissipation potentials should be changed to:52with $n'=1/n$. The notation $\langle \bullet \rangle _+$ indicates the positive part, so that the elasticity domain is defined by $\langle z\rangle _+=0$.

We now deal with the *Prandtl-Reuss model with (linear) kinematic hardening*. For this model, the variable sets are complemented as:53with $\mathbb {X}$ the kinematic hardening variable (backstress tensor) and $\alpha \hspace{-7.5pt}{\alpha }$ the associated internal variable. The free energy is modified in:54and the von Mises yield function is rewritten as:55with *A* and *C* some positive constants.

The normality rule then yields the following constitutive relations which correspond to yielding and hardening laws:56with .

#### Implementation of the error estimation and adaptive strategy

**Fig. 25:**
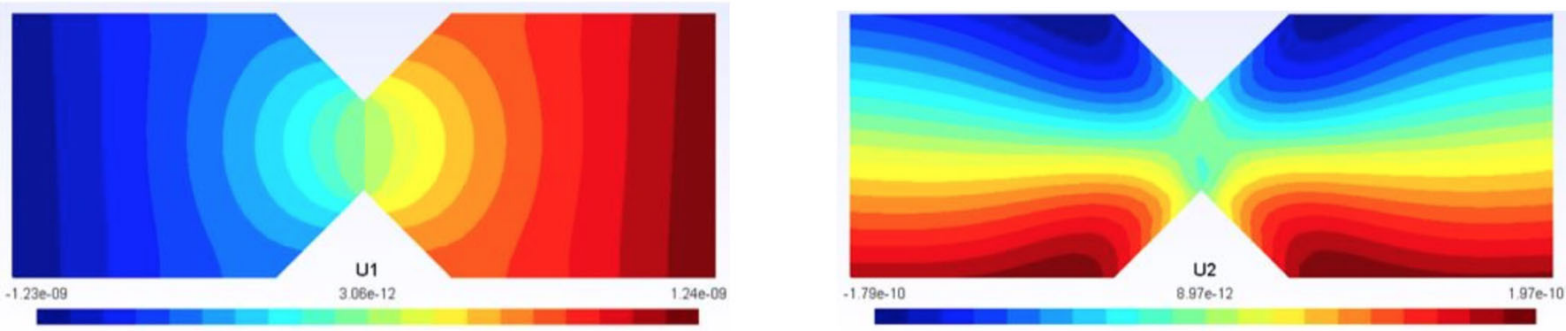
Admissible displacement field ${\hat{{\textbf{u}}}}^{sol}$ obtained from an elasticity model, with horizontal (left) and vertical (right) components

**Fig. 26:**
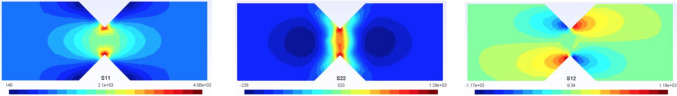
Admissible stress field obtained from an elasticity model, with ${\hat{\sigma }}^{sol}_{xx}$ (left), ${\hat{\sigma }}^{sol}_{yy}$ (center), and ${\hat{\sigma }}^{sol}_{xy}$ (right) components

We first start with a purely linear elastic model, and we try to identify the Young modulus *E* from the set of measurements. We show in Figs. 25 and 26 the optimal admissible displacement and stress fields obtained at the end of the process. The identified Young modulus value, obtained after 8 iterations of the mCRE algorithm, is $E=186$ GPa.

Next, an elastoplastic model with material behavior described by the *Prandtl-Reuss plastic law with linear isotropic hardening* is assumed. With this new model, the identified parameters are $E=206$ GPa, $R_0=154$ MPa, and $k=0.015$ MPa. When using the LATIN method in order to recover optimal admissible fields, about 15 sub-iterations are necessary for first iterations of the mCRE minimization, while between 2 to 5 sub-iterations are used for other minimization steps; this is due to the use of previously computed admissible fields to initialize the process (restart procedure explained in Sect. 2.4). The identification procedure is performed with 8 iterations to reach convergence.

Last, we change the model and consider the *Prandtl-Reuss plastic model with kinematic hardening* i.e., the one used to get measurements. The identified values are $E=203$ GPa, $R_0=164$ MPa, $A=0.59$ MPa$^{-1}$, and $C=45$ GPa, which are very close to the reference values (with relative errors of 1.5%, 2.5%, 6.3%, and 6.2%, respectively).

**Fig. 27:**
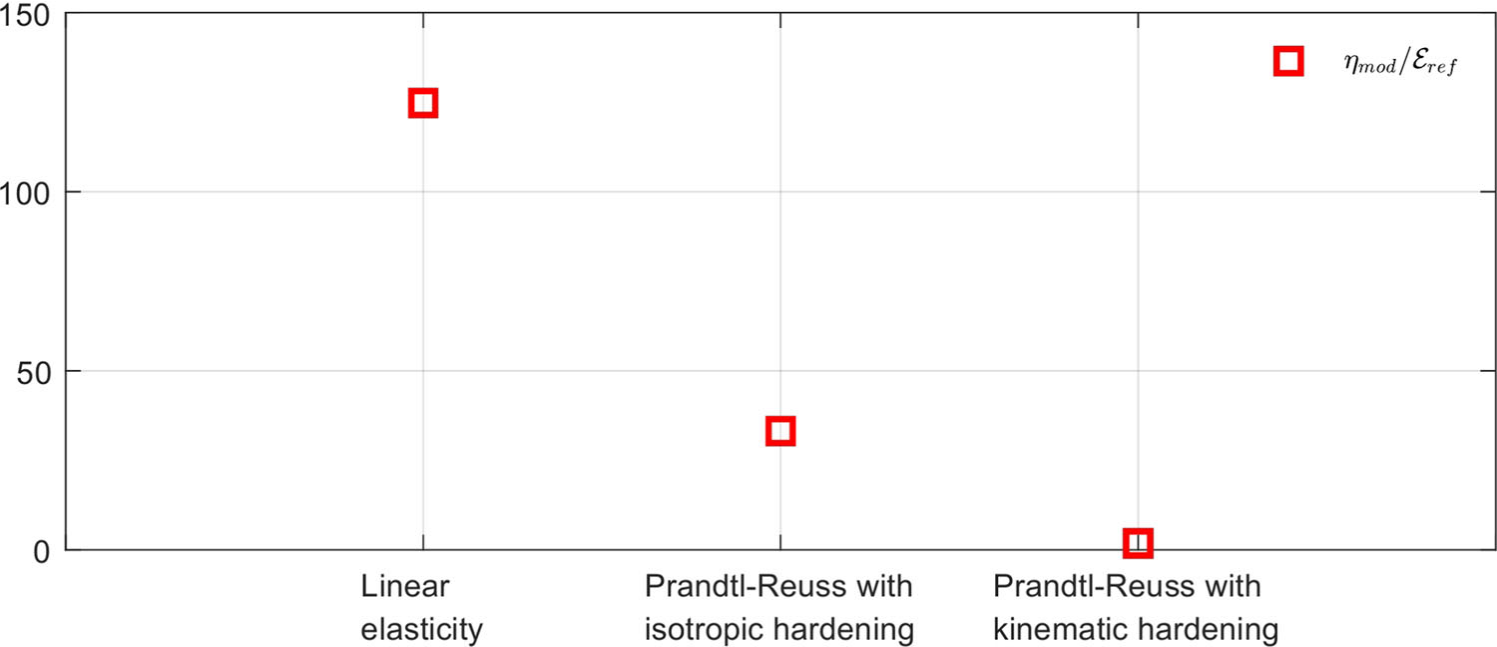
Relative indicator on modeling error obtained with the three considered models

In order to confirm the interest of the modeling error indicator $\eta _{mod}$ used in the adaptive model algorithm, we indicate in Fig. 27 the value of the relative indicator obtained at the end of the identification process from the three models considered. As expected, we observe that the indicator value is much larger than 1 when the model is not compatible with observations, while it is of the order of 1 when the reference model is selected.

### Application 3: identification from real data on a biaxially loaded cross-shaped specimen

**Fig. 28:**
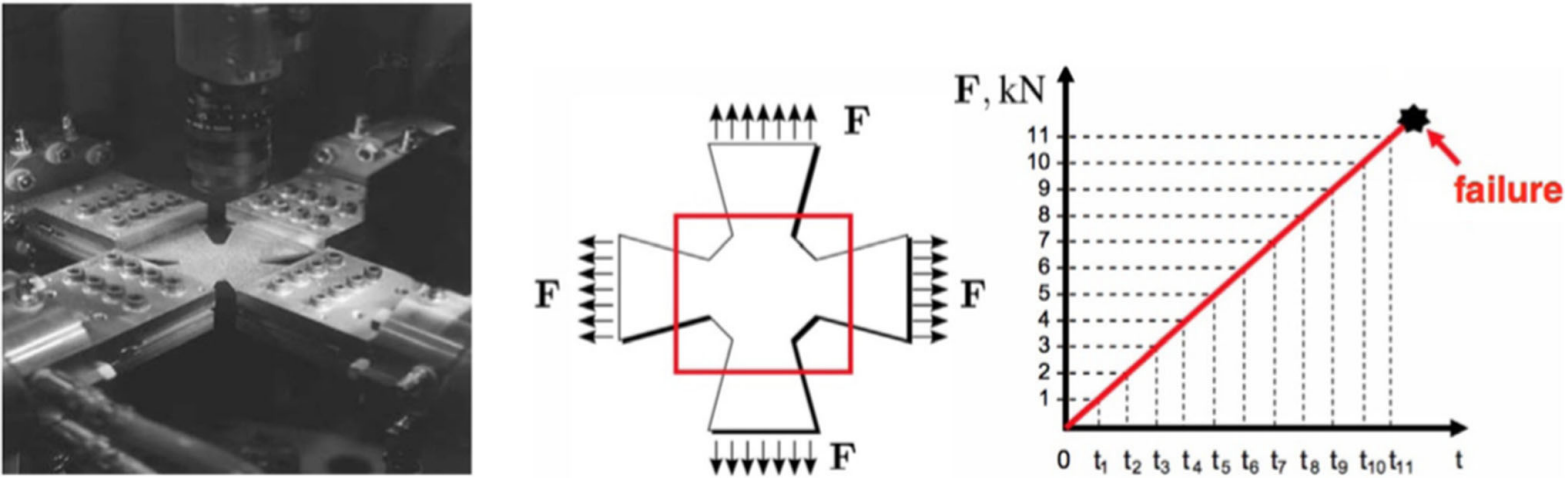
Biaxial tensile test of a cross-shaped composite specimen: experimental setup, measurement zone, and loading evolution

**Fig. 29:**
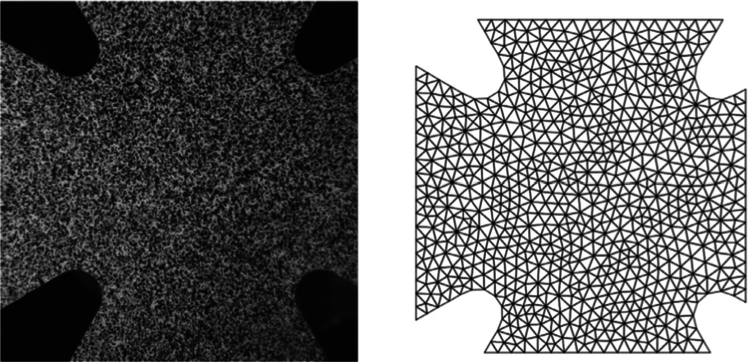
Reference picture for the DIC analysis and FEM discretization mesh for DIC

As a last illustration, we consider an identification experiment with a biaxial tensile test shown in Fig. 28. A cross-shaped specimen is biaxially loaded in a multiaxial testing machine. The studied material is made of a vinylester matrix reinforced by glass fibers. The quasi-uniform distribution of fiber orientations leads to an isotropic elastic behavior prior to heterogeneous matrix cracking and fiber breakage. We also consider experimental data in terms of full-field measurements obtained from quantitative imaging by means of DIC. The measurement zone is indicated with the red rectangle in Fig. 28; the experimental displacement field ${\textbf{u}}_{obs}$ is measured by taking images with a definition of 1008 $\times$ 1016 pixels at the various loading steps. The reference picture for the DIC analysis is shown in Fig. 29; the DIC mesh is made of 781 nodes, also corresponding to the simulation mesh in the mCRE-based identification strategy.

**Fig. 30:**
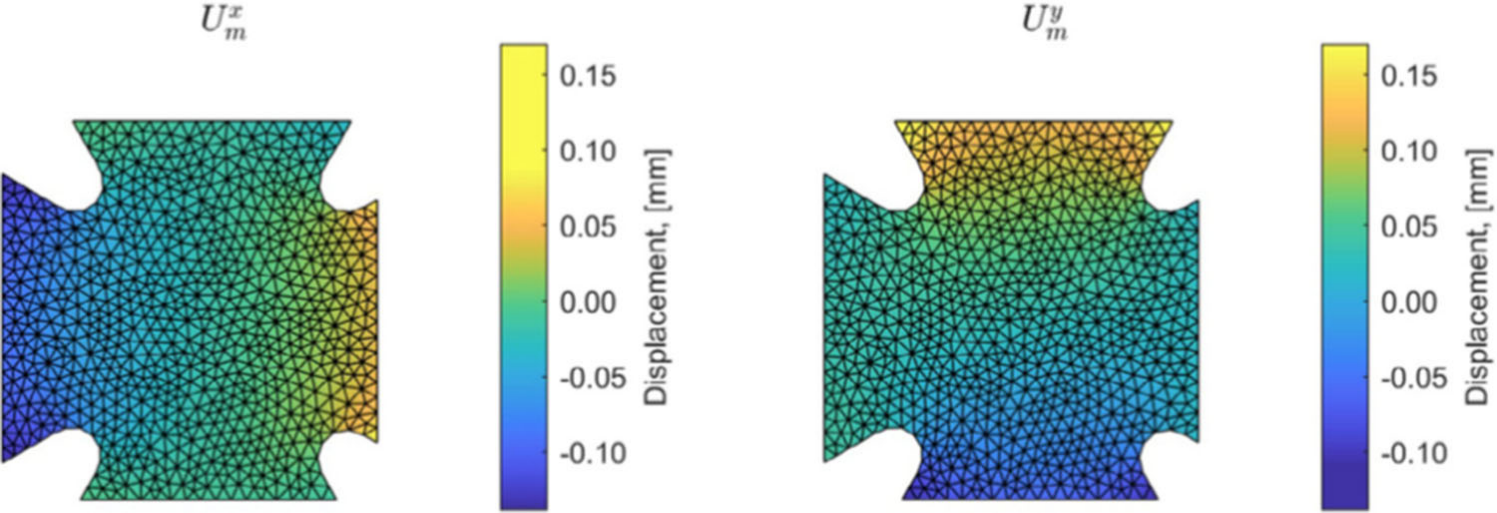
Horizontal and vertical components of the measured displacement field

**Fig. 31:**
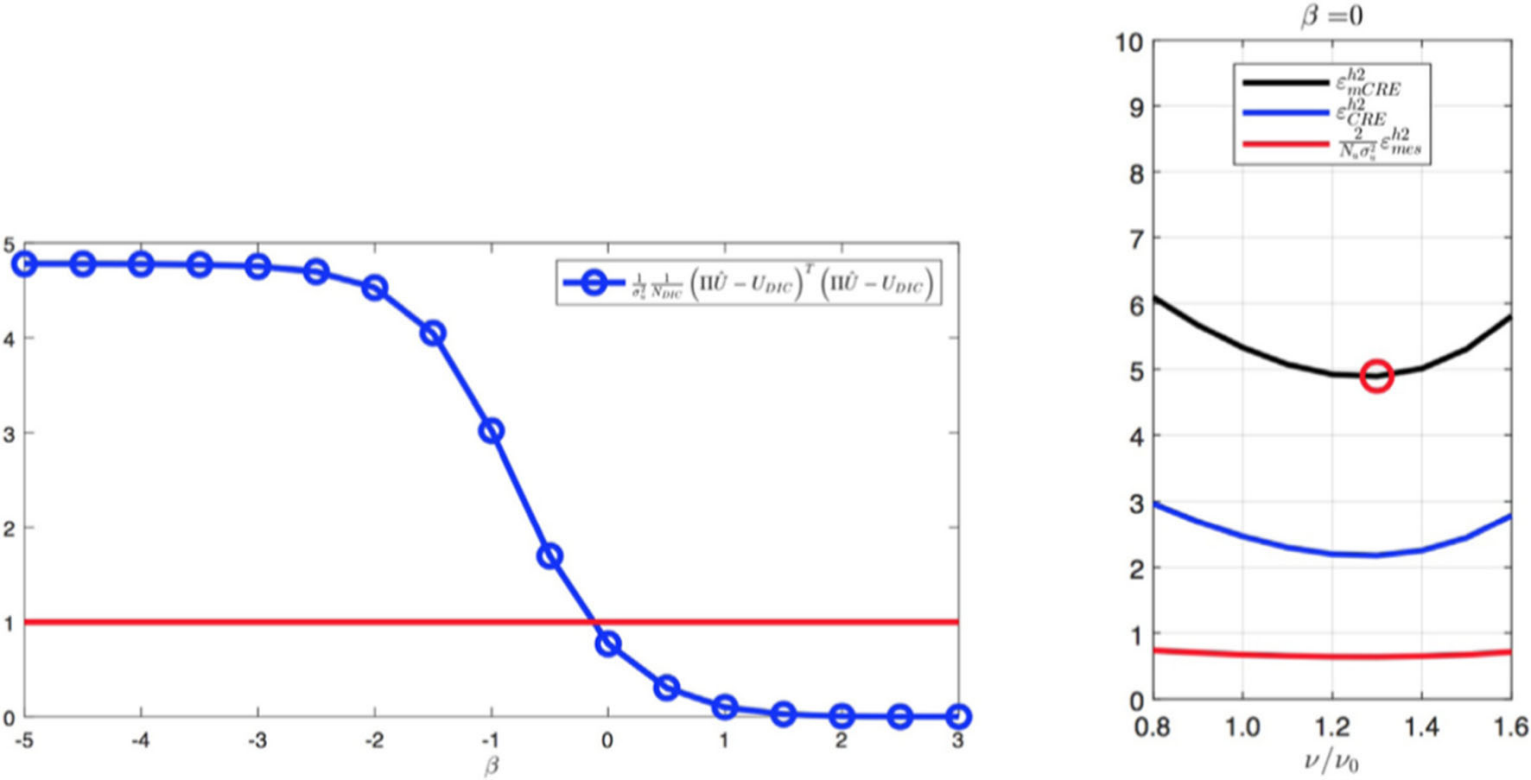
Evolution of the model-measurement mismatch term as a function of $\beta$, and evolutions of the mCRE functional and its two components as a function of $\nu$ for $\beta =0$

**Fig. 32:**
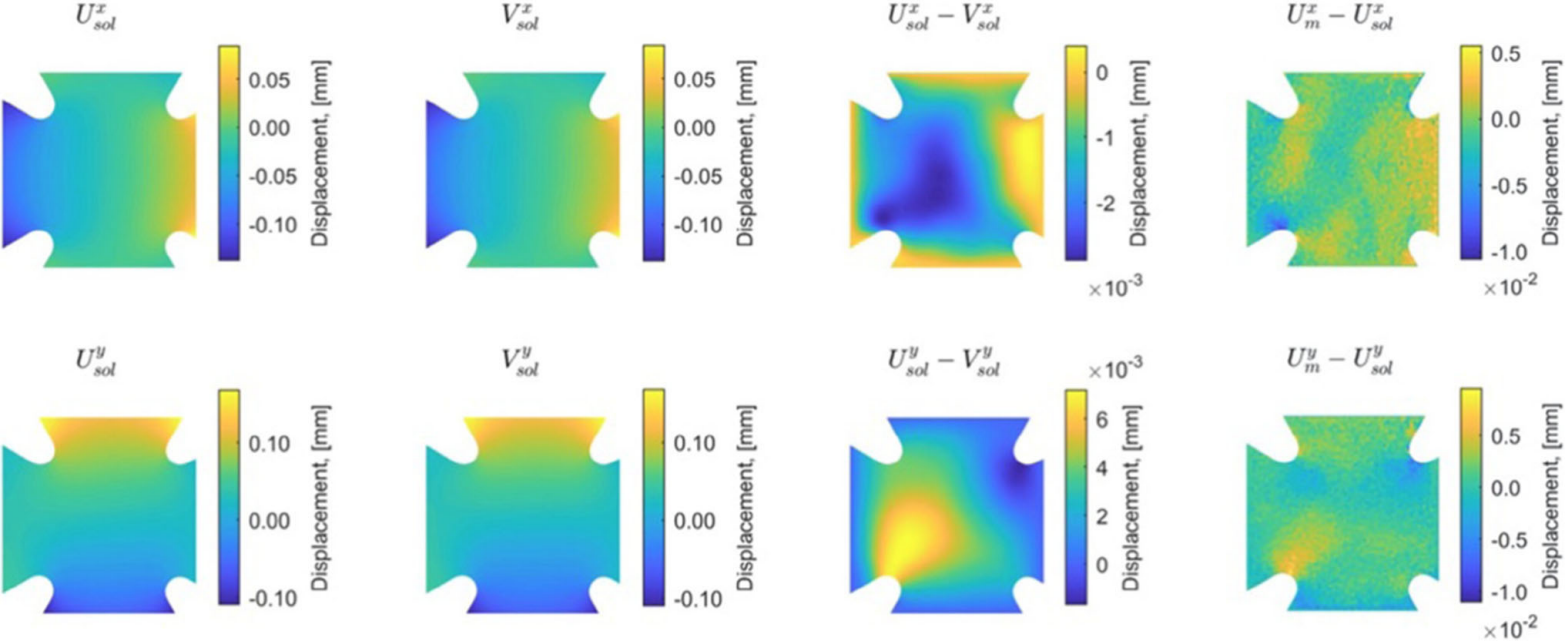
Spatial representation of admissible fields ${\hat{{\textbf{u}}}}$, ${\hat{{\textbf{v}}}}$, their difference, and ${\textbf{u}}_{obs}-{\hat{{\textbf{u}}}}$

Placing first in the linear elasticity regime of the composite specimen (at time $t=t_5$ that is with a loading $F=5$ kN), we wish to identify the Poisson ratio $\nu$ from mCRE and experimental information. The displacement field obtained from DIC is shown in Fig. 30. In order to implement the Morozov principle, we again define the weighting factor as $\alpha = 10^\beta .{\textbf{U}}_0^T\mathbb {K}_0{\textbf{U}}_0$ where ${\textbf{U}}_0$ and $\mathbb {K}_0$ are discretized displacement field and stiffness matrix associated with a reference solution. The obtained Morozov curve is shown in Fig. 31; it indicates that the optimal weight value is here about $\beta =0$. For this value, the evolution of the mCRE cost function and its two components is displayed in Fig. 31; the attractive convexity property can be clearly observed. The identified value is $\nu _{opt}/\nu _0 = 1.24$. We represent in Fig. 32 the corresponding optimal admissible fields. Large differences between kinematic and static fields in the bottom left corner indicate that damage starts initiating there, as confirmed by experimental observations. We also show the comparison between the kinematically admissible field and the measured displacement field.

We now apply the identification with mCRE in the nonlinear regime of the composite specimen, in order to implement the model selection strategy. The CRE term of the mCRE functional is then post-processed to drive the model complexity, as described in Sect. 3. We show below the relative value $\eta _{mod}/\mathcal {E}_{ref}$ of this CRE term for various model classes with increasing relevance with regards to the DIC measurements. This indicates that an elastoplastic model is quite relevant to explain the noisy observations, even though it is not perfect (Table 1).

## Conclusions and prospects

In this research work, on the basis of analysis of different error sources showed up in the process of parameter identification, we designed a general methodology around the duality-based (m)CRE concept in order to assess and master the quality of the computational model used. The methodology leans on dedicated error indicators which are employed as quantitative information to evaluate the quality of the model class and the mesh with regards to experimental information. These indicators are further used to drive an adaptive strategy that selects the appropriate model and discretization compatible with noisy data coming from full-field measurements. The proposed methodology was developed in details in the linear elasticity case, with insights for its direct extension to nonlinear dissipative constitutive laws (from thermodynamics bases and the Legendre-Fenchel duality), and with adjunction of PGD model reduction tools to enhance numerical efficiency. It was illustrated with several numerical experiments involving linear or nonlinear material behaviors. These confirmed that by using the indicators, a relevant identification process can be conducted with optimized complexity.

In practical engineering situations, data assimilation is very demanding in particular with complex highly nonlinear models, which is a limitation for real-time applications. The proposed work that promotes a sound and dynamical management of computing resources for effective identification thus opens up new avenues to address such real-time applications; this is of particular interest with emerging applications e.g., online monitoring of engineering systems by means of digital twins and feedback loop, which require fast diagnosis and prognosis in the framework of Dynamic Data-Driven Applications Systems (DDDAS) [23]. More broadly, the proposed work strengthens the link between material modeling, numerical methods, and experimental techniques, so that it may be useful for various scientific communities wishing to perform at best modeling and simulation in association with experimental data (with complex material descriptions, management of large experimental data, etc.).

Several prospects to this work can be listed. First, it would be fruitful to apply the proposed methodology on three-dimensional problems, e.g. with full-field measurements obtained from DVC/tomography, and on specific complex engineering problems such as damage problems (with adaptivity of the damage field representation) or multiscale material modeling (when *a priori* information on the microstructure is known). Also, when dealing with sequential data assimilation, the proposed approach could be inserted into the mDKF strategy introduced in [30, 67] and coupling mCRE and Kalman filtering.

Second, when dealing with parameter fields, it would be intersecting to analyze the interest of an additional sparse regularization (with Lasso regression) in the mCRE functional, as initially investigated in [33, 44], of the multiscale approach proposed with mCRE in [49], or of clustering techniques developed in [31].

**Table 1:** Relative CRE value for various model classes

Model class	Relative CRE value
Isotropic elasticity	17.56
Orthotropic elasticity	3.27
Elasto-plasticity (Prandtl-Reuss)	2.29

Eventually, a fundamental aspect which needs to be further analyzed is in the practical choice of a mathematical model. Here, we searched a model class among a given manifold, but it seems more powerful to have the appropriate model class freely learnt from AI (machine learning) tools, with less empiricism. This refers to the wide and recent bibliography on the data-driven learning of constitutive laws, using physics-informed or physics-augmented neural networks (NNs) (see [43, 50, 53, 69, 90] to list a few references). In this framework, the so-called NN-mCRE approach was investigated in [10, 11] for unsupervised learning of state and evolution laws i.e., of thermodynamic potentials $(\psi ,\varphi )$, with input convex neural networks (ICNNs) and a mCRE-based loss function. It could be used with the proposed adaptive strategy to conduct at best the data-based model enrichment.

All the previously mentioned topics will be addressed in forthcoming research works.
